# Fifteen Years of Wireless Sensors for Balance Assessment in Neurological Disorders

**DOI:** 10.3390/s20113247

**Published:** 2020-06-07

**Authors:** Alessandro Zampogna, Ilaria Mileti, Eduardo Palermo, Claudia Celletti, Marco Paoloni, Alessandro Manoni, Ivan Mazzetta, Gloria Dalla Costa, Carlos Pérez-López, Filippo Camerota, Letizia Leocani, Joan Cabestany, Fernanda Irrera, Antonio Suppa

**Affiliations:** 1 Department of Human Neurosciences, Sapienza University of Rome, 00185 Rome, Italy; alessandro.zampogna@uniroma1.it; 2 Department of Mechanical and Aerospace Engineering, Sapienza University of Rome, 00184 Rome, Italy; ilaria.mileti@uniroma1.it (I.M.); eduardo.palermo@uniroma1.it (E.P.); 3 Department of Physical Medicine and Rehabilitation, Sapienza University of Rome, 00161 Rome, Italy; clacelletti@gmail.com (C.C.); marco.paoloni@uniroma1.it (M.P.); filippo.camerota@uniroma1.it (F.C.); 4 Department of Information Engineering, Electronics and Telecommunications, Sapienza University of Rome, 00184 Rome, Italy; alessandro.manoni@uniroma1.it (A.M.); ivan.mazzetta@uniroma1.it (I.M.); fernanda.irrera@uniroma1.it (F.I.); 5 Department of Neurorehabilitation and Experimental Neurophysiology Unit, INSPE-Institute of Experimental Neurology, University Vita-Salute and Hospital San Raffaele, 20132 Milan, Italy; dallacosta.gloria@hsr.it (G.D.C.); leocani.letizia@hsr.it (L.L.); 6 Technical Research Centre for Dependency Care and Autonomous Living (CETpD), Universitat Politècnica de Catalunya, Vilanova I la Geltrú, 08800 Barcelona, Spain; carlos.perez-lopez@upc.edu (C.P.-L.); joan.cabestany@upc.edu (J.C.); 7 Sense4Care, 08940 Cornellà de Llobregat, Spain; 8 IRCCS Neuromed, 86077 Pozzilli (IS), Italy

**Keywords:** wireless sensors, wearables, balance, posturography, Alzheimer’s disease, Parkinson’s disease, multiple sclerosis, cerebellar ataxia, stroke, vestibular syndrome

## Abstract

Balance impairment is a major mechanism behind falling along with environmental hazards. Under physiological conditions, ageing leads to a progressive decline in balance control per se. Moreover, various neurological disorders further increase the risk of falls by deteriorating specific nervous system functions contributing to balance. Over the last 15 years, significant advancements in technology have provided wearable solutions for balance evaluation and the management of postural instability in patients with neurological disorders. This narrative review aims to address the topic of balance and wireless sensors in several neurological disorders, including Alzheimer’s disease, Parkinson’s disease, multiple sclerosis, stroke, and other neurodegenerative and acute clinical syndromes. The review discusses the physiological and pathophysiological bases of balance in neurological disorders as well as the traditional and innovative instruments currently available for balance assessment. The technical and clinical perspectives of wearable technologies, as well as current challenges in the field of teleneurology, are also examined.

## 1. Introduction

Countries are globally experiencing a demographic shift in the distribution of the population towards older ages [1] and every year up to 35% of people aged 65 and over fall, often requiring hospital admission after mild to severe injuries [2]. Falls account for 40% of all injury-related deaths [2], and even when non-fatal, commonly cause a “post-fall syndrome”, a psychomotor regression condition responsible for psychological, postural and gait dysfunction in elderly [3]. In terms of the economic burden of falls, in 2015 the estimated medical costs attributable to fatal and non-fatal falls increased to 50 billion dollars in the United States [4]. Falls represent a major public health concern and have an enormous economic impact on society, thus requiring the development of effective strategies to prevent underlying causes. Among these, balance impairment is one of the leading determinants of falls along with ecological factors, such as environmental hazards [5]. Ageing significantly impacts on postural ability due to age-related changes in the sensorimotor and cognitive function [6]. Moreover, balance impairment frequently affects patients with neurological disorders who are twice as likely to fall compared to an age-matched healthy population [7]. 

To date, a history of falls is the strongest predictor of future falls [8,9], thus underscoring the need for predictive measures to determine early preventive interventions. However, clinical assessment is subjective and is not sensitive enough to identify early balance control dysfunction [10]. Conversely, traditional laboratory evaluation, including posturography through force platforms and optoelectronic systems, is objective and sensitive enough to identify subtle abnormalities but does not always reflect real-life situations. Over the last 15 years, advancements in healthcare technology have allowed analysing physiological measures of motor and non-motor behaviour objectively and unobtrusively [11]. Indeed, the availability of wearable devices has opened to the instrumental evaluation of clinical phenomena in free-living conditions. Accordingly, several authors have made a great effort to use wireless sensors in the study of balance impairment in patients with neurological disorders, thus offering new solutions for diagnosis and rehabilitation [12].

Despite several previous reviews discussing specific technical or clinical aspects of balance assessment through wearables, this narrative review aims to discuss the whole topic of balance evaluation, through wireless sensors, in patients with neurological disorders. Accordingly, in this review, we first introduce the physiology and pathophysiology of balance, including the main mechanisms underlying postural dysfunction in several neurological disorders, and report clinical tools commonly used for balance assessment. We then summarise the instrumental assessment of balance, including static and dynamic posturography. Moreover, we analyse wearable technologies available for balance assessment in neurological disorders. Finally, we speculate about prospects and challenges of wireless sensors for balance assessment in teleneurology and telerehabilitation.

## 2. Physiology and Pathophysiology of Balance 

Balance is the ability to maintain body orientation in space under static and dynamic conditions [13], respectively intended as postural stability at rest and in response to active movement or external perturbations. Over the course of evolution, the complexity of this function greatly increased with the acquisition of vertical posture and bipedalism in humans, representing the main transformation in primates [14]. A composite sensorimotor-control system based on a closed-loop circuit dynamically coordinates body segments according to environmental hazards through feedback and feed-forward strategies [15]. 

**Figure 1:**
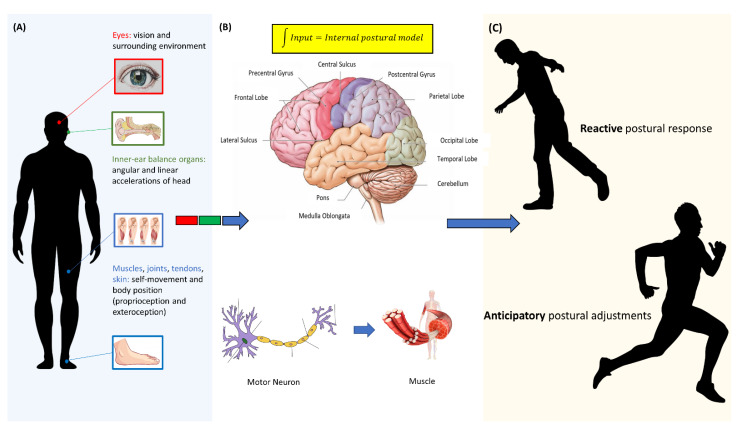
Physiology of balance. (**A**) The visual system provides information on the surrounding environment; the vestibular system, consisting of the two inner-ear balance organs and several nervous structures (nerves and central nuclei), encodes angular and linear accelerations of the head to support the clear vision and balance control via rapid eye movements (vestibulo-ocular reflexes) and postural reflexes (vestibulo-spinal reflexes); the somatosensory system senses self-movement and body position through specialised sensory receptors located in the muscles (muscle spindles), joints (Ruffini endings, Pacinian corpuscles, and Golgi-like receptors), tendons (Golgi tendon organs), and skin (Merkel cells, Ruffini endings, Meissner corpuscles, and Pacinian corpuscles) [20,21]. (**B**) Multisensory signals from visual, vestibular and somatosensory receptors are integrated in the central nervous system to provide an internal postural model and in turn, descending motor commands to muscles. (**C**) Reactive postural strategies and anticipatory postural adjustments allow balance control under environmental circumstances (e.g., external postural perturbations) and motor initiative (e.g., voluntary movement), respectively.

The central nervous system oversees balance maintenance by integrating sensory inputs from the peripheral nervous system (e.g., receptors and nerves) and motor outputs to the musculoskeletal system [15,16] (Figure 1). Brainstem nuclei, along with basal ganglia, the cerebellum, and other subcortical structures (e.g., thalamus) play crucial roles in the integration of sensory cues from the somatosensory, vestibular, and visual systems, which continuously provide an overall representation of body movement, acceleration, and position in space [15,17] (Figure 1A). By encoding an internal postural model based on reciprocal connections with the parietal cortex, the cerebellum contributes to dynamic balance control through postural responses that serve as an error-correction mechanism [16] (Figure 1B). Finally, the cerebral cortex oversees attentional and visuospatial balance requirements and manages anticipatory postural adjustments (APAs) before and during voluntary movements [18]. Cognitive-motor processes are responsible for postural optimisation based on prior experience, current context, and learning through long-latency components of postural responses [19]. 

The main goal of physiological mechanisms underlying balance control is the maintenance of postural stability by managing the spatio-temporal relationship between the body’s centre of mass (COM) and base of support (BOS) [22]. While reactive postural responses compensate for unexpected external perturbations, proactive postural responses allow balance control under expected external perturbations or self-produced balance disturbances through a motor prediction strategy [22]. When an external balance perturbation occurs, different postural strategies are adopted to maintain the COM projection within the BOS. Indeed, minor postural perturbations are usually counteracted by corrective strategies involving body rotations around the ankle (ankle strategy) or hip (hip strategy) that move the COM projection. Conversely, major postural disturbances require a broadening or displacement of the BOS in order to maintain the COM projection within the BOS (protective strategy) [22] (Figure 1C). 

Three main pathophysiological mechanisms are responsible for balance dysfunction: (i) abnormal acquisition, transmission, or perception of sensory signals (Figure 1A); (ii) abnormal sensorimotor integration and motor planning (Figure 1B); (iii) impaired transmission of motor output or musculoskeletal system damage [23] (Figure 1B,C). In patients with impaired afferent sensory information (e.g., somatosensory, vestibular or visual inputs), balance control requires compensatory strategies including attentional resources [24] and sensory reweighting [25]. 

Ageing is commonly associated with a progressive loss of sensorimotor function, including structural and functional changes in the somatosensory, visual, and vestibular systems, along with a decline in central neural processing and muscle strength [6]. Accordingly, ageing leads to slower reaction times and reduced limits of stability, thus worsening balance control mainly under cognitive loads and unexpected postural perturbations [6,26]. 

Patients with neurological disorders may manifest balance dysfunction as a result of impairment of at least one physiological component responsible for balance control significantly increasing the risk of falls compared to age-matched healthy subjects [7]. Pathophysiological mechanisms leading to balance impairment in various neurological disorders are summarized in Table 1 along with the main nervous system structures underpinning postural dysfunction. Understanding the physiological mechanisms underlying balance control in humans is the necessary background to measure balance objectively, through conventional as well as wearable technologies, in patients with neurological disorders.

## 3. Clinical Assessment of Balance

The clinical assessment aims at recognizing balance impairment and identifying possible underlying causes [63]. Neurological examination routinely involves several clinical manoeuvres, including the Romberg’s test [64], the pull test [65], and the tandem gait test [66], designed to examine individual balance performance qualitatively (Table 2). In addition to these clinical manoeuvres, several standardized scales and tests provide a semiquantitative evaluation of balance (Table 2). A secondary task during motor performance (i.e., dual task) is commonly used to assess the involvement of cognitive function in balance control. 

When considering the clinical assessment of balance, several issues should be taken into account. First, the clinical assessment unlikely detects early postural abnormalities since it identifies balance impairment when significant pathological changes in the nervous system have already occurred. Second, the clinical assessment provides qualitative rather than quantitative evaluations of postural ability, thus representing a subjective tool. Third, standardised clinical scales or indices, such as the Berg balance scale [75] or the dynamic gait index [76], are semiquantitative evaluations of balance, but are time-consuming and suffer from floor and ceiling effects. Lastly, the clinical setting usually involves rather predictable environments with poor ecological value. As a result, evaluation through instrumental tools, such as wearable sensors, would contribute to providing more sensitive, objective, multidimensional, long-term and ecological measures.

## 4. Static and Dynamic Posturography 

Posturography refers to the instrumental assessment of balance [77,78,79] under static or dynamic conditions [80,81]. Static posturography examines body postural sway while subjects maintain a static stance on a non-movable surface [79,81]. During the upright stance, the human body can be considered an unstable system in which force gravity and body inertia generate torques to be balanced [82]. Indeed, the vertical projection of the whole body mass constantly varies over time, deviating from the ankle joint centre of rotation [83]. Human standing balance can be represented by a reduced number of joints resembling an unstable single-link inverted pendulum [84]. 

Unlike static evaluation, dynamic posturography includes several postural tests and ad-hoc instruments designed to assess balance under experimentally-induced external perturbations [85]. External disturbances are often designed to simulate environmental hazards occurring in daily activities including a set of visual and motor challenges [85,86]. Postural responses to external perturbations can be assessed by a non-motorised movable platform, such as the Biomechanical Ankle Platform System [87], or more complex commercial robotic systems, such as the Equitest system (Neurocom International, Clackmas, OR, USA) [85], the Balance Master (Micromedical Technologies, Chatham, IL, USA) [88], or Caren (Motek, Amsterdam, the Netherlands) [89].

Several non-commercial robotic platforms have been recently designed to provide various patterns of mechanical perturbation [90,91,92,93,94]. Common approaches include unidirectional [95] or multidirectional [85,96,97] disturbances, such as rotational [93,98,99,100,101] and translational perturbations [96,102,103,104], or forces applied to specific body segments [105,106]. Abrupt perturbations allow the examination of reactive postural responses, whereas continuous and oscillatory perturbations are used for the assessment of anticipatory postural strategies [101,102,104,107,108]. Postural perturbations can be also defined as predictable or unpredictable according to the subject’s awareness. The predictability/unpredictability of a specific perturbation allows the experimental investigation of reactive or anticipatory postural strategies [105,106,109]. Mechanical perturbations are often merged with visual, vestibular, and proprioceptive disturbances such as visual scene movements, imposed head accelerations, galvanic vestibular stimulation, and tendon vibration [81,85,89,110,111]. The most common tests used are the Sensory Organization Test (SOT) [112], the Motor Control Test (MCT) [113], and the Adaptation Test (AT) [81]. In the SOT, subjects are elicited through visual, vestibular, and proprioceptive modifications of the support surface and visual surroundings to create sensory conflict conditions. The MCT consists of antero-posterior perturbations at different intensity levels, while in the AT subjects experience toes-up and toes-down rotations.

Several biomechanical parameters quantify balance dysfunction [114,115] by referring to two main variables: the centre of pressure (COP) and COM [116]. The COP is the application point of the total ground reaction force vector, whereas the COM refers to the average position in 3D space of all body segment positions according to their specific masses [116]. COM can be considered representative of the movements of the entire human body [116]. Several indices considering acceleration, velocity, displacement of single or multiple body segments, joint angles, and muscle activity can be measured using both traditional and wearable instrumentation (Table 3).

Overall, classical laboratory posturography through force plates and optoelectronic systems provides reliable, accurate, and comprehensive measurements for balance assessment. However, these techniques are generally expensive, encumbering, and also require supervised settings as well as technical expertise, thus precluding their use for long-term monitoring in daily life situations. Accordingly, current research on posturography has recently moved on wearable technologies [132,133,134,135,136,137] possibly providing objective, long-term and free-living monitoring of postural ability at a negligible cost.

## 5. Wearable Technologies

**Figure 2:**
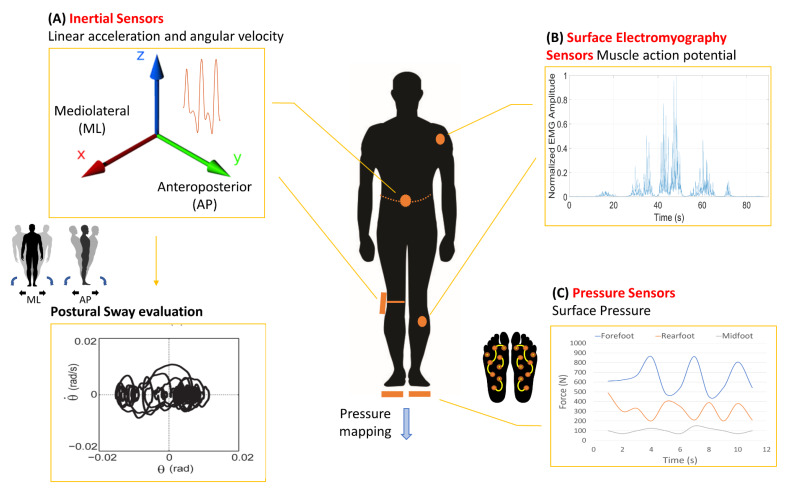
Wearable technologies. Three main types of wireless sensors are available for motion analysis and balance assessment, including mechanical (inertial and pressure sensors) (**A**,**C**) and physiological (surface electromyography sensors) (**B**) wearable devices. AP: antero-posterior; ML: medio-lateral.

Recent advances in microelectronics have led to the production of small flexible sensors, even integrated into clothing (“e-textile”) [138], thus making wearable devices suitable for free-living applications [139]. To date, the main wearable technologies available for balance assessment include mechanical devices, such as inertial and pressure sensors, and physiological devices, such as surface electromyography sensors (sEMG) (Figure 2). Wireless inertial sensors are the most used solution in wearable systems and have been widely adopted for balance and gait assessment [115,140,141,142]. Half of the previous studies used commercial inertial measurement unit (IMU) sensors including triaxial accelerometers and gyroscopes, and half adopted stand-alone accelerometers [143] or gyroscopes [144]. The combination of triaxial accelerometers, triaxial gyroscopes and magnetometers compose magnetic and inertial measurement units. Sensor placement depends on the specific postural task under investigation [115]. For instance, wearable sensors can be placed over the waist or trunk in order to measure postural sway and trunk acceleration. Other possible body locations include the lower limbs, sternum, upper limbs and forehead. Triaxial sensors can capture spatio-temporal and 3D kinematic data including joint and segment angles [145,146,147]. Overall, the combination of accelerometers, gyroscopes, and magnetometers provides accurate information on body spatial orientation and motion (Figure 2A). Besides inertial devices, wearable sEMG sensors evaluate specific patterns of muscle activation during static and dynamic postural perturbations. sEMG, therefore, allows a better understanding of physiological mechanisms responsible for balance control [148,149] (Figure 2B). Lastly, wearable pressure sensors are instrumented insoles placed or integrated into the shoe to measure pressure changes between the foot and ground [150]. The accuracy of this discrete sensor system is comparable to non-wearable technologies such as the laboratory force platform (Figure 2C). In addition to mechanical and physiological devices, there are wearable sensors able to continuously monitor the concentration of specific biochemical markers in biofluids, through miniaturized and flexible devices [151]. These innovative sensors would open to interesting prospects also referring to the assessment of balance. For instance, monitoring L-Dopa or dopamine concentration by microneedle patches would be a helpful tool to correlate postural ability with dopaminergic treatments in patients with Parkinson’s disease [152,153]. Currently, several wearable sensors, mostly including inertial devices, are available on the market for approved clinical use in balance assessment [154], also including self-adhesive biosensors (for further details see www.clinicaltrials.gov).

The large volume of data produced by wearable sensors requires the development of specialised algorithms and machine learning algorithms to select clinically-valuable measures [138]. Owing to the considerable processing capacity of wearable devices, embedded algorithmic sets can be used for the online and remote execution, but at the expense of the battery charge duration. To optimise the performance of these algorithms in recognising clinical phenomena, a common approach leverages the so-called “sensor fusion”, which consists of the combination of sensory data and signals derived from distinct sources so that the resulting information is more accurate (e.g., integration of inertial and electromyography signals) [155]. Accordingly, the emerging trends in wearables are moving towards the design of integrated sensors, including devices composed of IMUs and sEMG [148], to be user-friendly, waterproof and unobtrusive. Table 4 summarises the strengths, limitations and challenges of each type of wireless sensors currently used for balance assessment. Moreover, Table S1 reports all the previously published reviews on balance assessment through wearable devices in healthy subjects and patients affected by various medical conditions. 

## 6. Literature Research Strategy and Criteria

Literature research of studies investigating balance impairment through the use of wireless sensors in neurological disorders was performed using the following databases: MEDLINE, Scopus, PubMed, Web of Science, EMBASE and the Cochrane Library. Literature criteria included the following terms: “wireless sensors” OR “wearables” OR “inertial measurement unit” OR “surface electromyography” OR “pressure sensors” AND “neurological disorders” OR “Alzheimer’s disease” OR “stroke” OR “Parkinson’s disease” OR “multiple sclerosis” OR “vestibular disorders” OR “cerebellar ataxia” OR “traumatic brain injury” OR “Huntington’s disease” OR “neuropathy” AND “balance” OR “posturography” OR “postural control.” Eligible studies were experimental studies published from January 2005 to March 2020, examining balance through wireless sensors in patients suffering from the above reported neurological disorders. The reference lists of retrieved articles were also manually searched for additional studies. Reviews, reports, conference proceedings, and articles in languages other than English were not considered in the evaluation of eligible studies.

## 7. Wearable Technologies in Neurological Disorders

Previous studies using wearable sensors have investigated balance impairment in Parkinson’s disease [114,122,124,125,156,157,158,159,160,161,162,163,164,165,166,167,168], multiple sclerosis [118,146,169,170,171,172,173,174,175,176,177], stroke [52,178,179,180,181,182,183,184], traumatic brain injuries [123,126,185,186,187,188,189], cerebellar ataxia [130,190,191,192,193,194,195], vestibular syndromes [196,197,198,199], neuropathies [199,200,201], Alzheimer’s disease [32,202,203], and Huntington’s disease [46,204]. Most of these studies have compared patients affected by neurological disorders with healthy subjects. However, a minority of authors [52,167,176,178,180,187] have analysed postural ability only in a group of patients with neurological disorders without including a control group.

Concerning the type of sensors used for balance assessment, most of the existing studies have applied inertial devices, primarily accelerometers and gyroscopes. Several authors [46,162,163,183,184] have even used inertial sensors installed in common tablet computers and smartphones. Conversely, no authors have used pressure sensors, while only a few have adopted wireless sEMG sensors [166,167,168] to analyse balance impairment in patients with Parkinson’s disease. Strengths and limitations of each type of sensor are shown in Table 4. Each type of sensor technology would be implemented by addressing some challenges, including the elaboration of new algorithms, the development of implantable EMG tools and, finally, the use of unobtrusive “e-textile” devices (see Table 4). Also, future studies would benefit from the integration of various sensor technologies (i.e., sensor fusion) to optimize the measure of balance dysfunction in patients with neurological disorders. 

Regarding the number and body location of sensors, authors have used 1 to 8 inertial devices and multiple body segments, including the upper (10 studies) and lower limbs (21 studies), head (1 study), trunk (18 studies), and waist (48 studies), depending on the static or dynamic postural task chosen for balance assessment. Indeed, some authors who investigated postural evaluation during gait (e.g., [122,146,161,169,172,175]) and instrumented versions of clinical tests, such as the push and release test [171] and the Fukuda Stepping Test [182], have usually applied more sensors than those evaluating static balance during upright stance (e.g., [52,114,125,157,158,163,177,183,184,185,188,191,192,193,194,199,203,204]. However, despite one study [204], all authors have included the lumbo-sacral region as the main location of inertial sensors for the analysis of postural sway, according to the COM position. Conversely, multiple sEMG sensors have been placed mainly on lower limbs to monitor muscle activity during postural perturbations [166,167,168]. The number of sensors and their placement on the body is a relevant issue for balance assessment, also requiring to consider a proper cost and energy-benefit analysis, as well as the efforts for patients and caregivers. The number of sensors to be used depends on the specific clinical phenomenon under investigation (e.g., postural sway for balance control) and the need for maintaining high-quality measurements, through appropriate sampling rate and estimated energy consumption. Indeed, though more informative, a high number of devices would be computationally demanding and expensive, as well as uncomfortable to be applied in a domestic environment.

Considering the accuracy of sensors in balance assessment, some authors [52,114,156,157,159,162,164,171,173,174,186,191,193,194,200] have compared wearable device measurements with those of standardised laboratory measurement systems, such as force plates and 3D motion-capture systems. These authors have agreed on the moderate or strong correlation between specific inertial indices (e.g., root mean square of acceleration time series [114], acceleration peaks of anticipatory postural adjustments [156,159], time to reach stability [171]) and COP or optical measures, thus suggesting an accurate performance of inertial wearable devices compared to standardised instrumentations in the laboratory. However, validation studies in unsupervised settings are warranted to further support the reliability of wireless sensors for balance assessment in domestic environments. 

Most authors [32,52,114,118,123,124,125,126,130,146,157,158,163,165,169,170,173,174,176,177,178,183,185,186,187,188,189,190,191,192,193,194,195,197,198,199,200,201,202,203,204] have performed a static balance evaluation by analysing maintenance of the upright stance with different amplitudes of the BOS (e.g., side-by-side, tandem, single-leg stance). These protocols have also included the assessment of sensory and cognitive contribution to balance control by removing visual and/or proprioceptive cues (e.g., closed eyes, foam surface) and by increasing cognitive load (e.g., dual-task). Moreover, a large number of authors [46,118,122,146,156,159,160,161,162,164,166,167,168,169,171,172,173,175,179,180,181,182,190,196] have investigated dynamic postural control, mostly through the use of walking tasks, instrumented versions of clinical tests (e.g., Timed-Up and Go, stand and walk, and push and release tests), and external or self-triggered postural perturbations. Although several authors [46,123,125,157,161,166,167,168,171,172,173,178,179,180,181,190,196,202] have assessed balance during tasks possibly reflecting daily postural challenges, all research protocols have been conducted in a laboratory setting. However, since supervised laboratory settings only partially reflect challenging “real-life” situations, these studies do not provide firm conclusions about the application of wireless sensors in a domestic environment.

**Figure 3:**
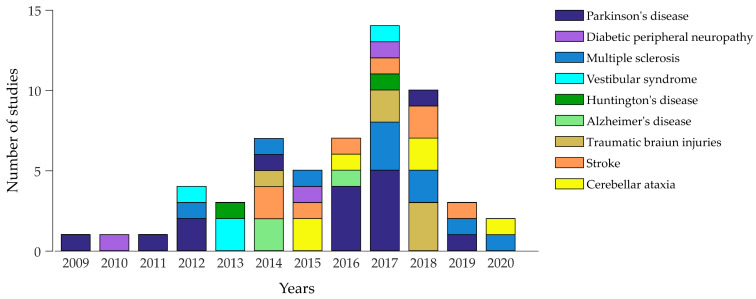
Trend of published studies on wireless sensors for balance assessment in neurological disorders. The figure includes studies reported in Table 5 and Table S2, based on the literature research through MEDLINE, Scopus, PubMed, Web of Science, EMBASE and the Cochrane Library (accessed on 31 March 2020). Note that the figure does not include a study published in 2005 [190] for graphical reasons.

Concerning biomechanical measures, previous studies have used filtered acceleration signals by inertial sensors to measure body sway in all the neurological disorders here considered, but have evaluated APAs during gait initiation only in patients with Parkinson’s disease. Overall, these measures have shown increased postural sway in patients with neurological disorders and decreased APAs during gait initiation in patients with Parkinson’s disease, as compared to age-matched healthy subjects. These parameters have also identified subclinical postural abnormalities (e.g., in vestibular syndromes) correlating with the amount of clinical disability [114,118,124,146,163,165,170,171,175,176,184,190,195]. A few authors [166,167,168] have measured muscle postural synergies with sEMG sensors in patients with Parkinson’s disease. Given that no studies have directly compared biomechanical indices in patients with different neurological disorders, it is unclear whether any of the measures may discriminate the various conditions. These findings overall have shown that wireless sensors can accurately quantify several kinematic measures, including the time and frequency COM dynamics [114,174,200], the 3-D trajectory of body sway angles [191], the joint range of motion [205], the stepping latency [171], and the APAs [159]. Conversely, the evaluation of kinetic measures, including the analysis of internal forces and moments acting on human joints, by wearable systems remains quite challenging [206]. Although the novel approach by wearables would help to partially overcome this issue with inertial and pressure sensors, inverse dynamics techniques, through motion capture systems and force platforms, are currently more suitable to achieve these measures. Moreover, to date, other dynamic variables, including the joint power and the energy cost of a movement, have not yet been evaluated by wearable sensors. Specifically concerning APAs, in addition to inertial measurements, wearable technologies would also allow long-term APAs recordings, through wearable sEMG, in more ecological environments. However, APAs recordings through wearable sEMG would require advanced algorithms for pattern recognition to achieve consistent observation. A further consideration concerns the generalizability to more ecological environments of behavioural measures observed in the laboratory setting. Unlike motor performance under “real-world” postural perturbations, experimental measures under a supervised laboratory setting would improve per se patients’ motor behaviour owing to unspecific and disease-unrelated factors, such as attentional and emotional aspects. The appropriate selection of a standardised measure for balance assessment would promote more consistent evaluation among the various neurological disorders. Table 5 provides an overall overview of the methodological approaches and findings from studies here examined. Also, a more detailed description of these studies is shown in Table S2. Finally, Figure 3 shows the positive trend of published studies on wireless sensors for balance assessment in the various neurological disorders.

## 8. Teleneurology and Telerehabilitation for Balance: Prospects and Challenges

Along with the ageing of the population, the prevalence of neurological disorders will also significantly increase in the next decades [207]. Accordingly, public health challenges will burden society and healthcare systems, which will face a heavy demand for the neurologic care of acute and chronic conditions. By allowing long-term monitoring for preventive and recovery strategies, wireless sensors will promote teleneurology and telerehabilitation and take some of the burden off of healthcare facilities.

Concerning the role of teleneurology for balance assessment through wireless sensors, so far, a few studies have addressed this topic in patients with neurological disorders. Nevertheless, several advantageous clinical prospects related to this issue should be considered. First, access to care for patients with balance impairment is quite challenging due to transportation difficulties and dependence on caregivers. Wireless sensors would be a sensitive and objective tool for the domestic measurement of balance control during the performance of validated instrumented tasks, such as maintenance of an upright stance. Moreover, other symptoms commonly associated with postural dysfunction, such as gait disorders [208], would also be measured, thus providing more detailed clinical information. Current evidence suggests that teleneurology promotes a reduction of patient and caregiver burden [209]. Second, medical visits in a hospital setting do not always reflect real-life situations, which commonly present insidious postural challenges. Therefore, the long-term monitoring of postural ability during common daily activities could provide ecological data on patient balance control in free-living conditions. This approach would help to identify early subclinical changes of balance, allow the objective assessment of fall risk and design individualised strategies for fall prevention (e.g., use of mobility aids and changes of environmental hazards). Third, the real-time identification of situations at high risk of falling would also allow patients to benefit from temporary preventive or rescue interventions. For instance, the detection of near-falls could be used for the automatic activation of protective tools, such as inflatable hip pads aimed to prevent fall-related injuries [210]. A further strategy would include the improvement of balance control by wearable-based sensory biofeedback, able to enhance patients’ awareness and in turn, prevent falls [211,212].

Along with fall prevention strategies, rehabilitation is the main therapeutic approach for improving balance in patients with neurological disorders. The main goal of rehabilitation is to enhance individual postural skills, supporting patient independence in ecological settings. To this aim, by using information and communication technologies, telerehabilitation would provide rehabilitative services directly at home [213] with similar effectiveness to conventional therapy [214]. Wireless sensors would allow monitoring of individual postural ability in a domestic environment, increasing adherence to the rehabilitative programme, and thus promoting tailored therapeutic approaches [215]. Moreover, wireless sensors would also support home-based interactive rehabilitation programmes by providing real-time feedback during unsupervised training. Nowadays, the increasing use of mobile phones and other technological tools in multiple aspects of daily life is promoting a widespread technological education in the general population, including the elderly. Accordingly, in the next decades, user-friendly wearables will be increasingly used to increase adherence to telerehabilitation strategies. Owing to remote and continuous evaluation by physicians and physical therapists, telerehabilitation would reduce the number of periodic hospital admissions. However, some initial education to patients and caregivers concerning wearables applications for therapeutic purposes is likely required. So far, several clinical trials have already adopted sensor-based measurements to objectively evaluate balance and its response to pharmacological as well as non-pharmacological interventions [216] (for further details see www.clinicaltrials.gov). However, only a few authors [216,217,218,219,220] have examined the effectiveness of sensor-based balance training in patients with neurological disorders. Furthermore, most of these studies involved a laboratory or clinical setting supervised by experienced staff [216]. Hence, to reach some firm conclusion, new randomised controlled trials should assess large samples of patients in ecological settings, including the domestic environment [216].

The main current challenge is the technological migration of wireless sensors from the laboratory setting to a domestic and unsupervised environment. The technological feasibility of sensor systems primarily depends on the variables to be measured as well as on the computing-capacity integrated into the wearables. Unlike conventional laboratory systems, the domestic use of wearable sensors would imply some limitations such as autonomy and interface capabilities (e.g., interaction with the user, communication with external devices and servers for information sharing). Concerning IMUs, challenges include the calculation capacity, which mainly depends on the running algorithms thus influencing the selection of a specific device, processing characteristics, memory capacity and communication protocol. Overall, the technological migration of wireless sensors from the laboratory setting to a domestic environment would benefit from the identification of standardized and accurate measures. To this aim, understanding the physiological and pathophysiological mechanisms underlying balance is the background for selecting, measuring and interpreting the specific postural variables to be assessed. Also, the improvement of communication between wearable sensors and external devices, as well as the implementation of standardized and low energy-consuming algorithms are additional limitations to overcome. To support this migration process, current commercialization efforts are reducing sensor dimensions to ensure the unobtrusiveness of the devices, though maintaining safety and accuracy standards. “Real-world” evidence aimed at monitoring balance disorders through wireless sensors in ecological settings (e.g., patients’ home or nursing home) will further clarify strengths and limitations in the telemedicine and telerehabilitation approaches. 

Several open questions remain when considering teleneurology and telerehabilitation approaches. To date, only a few randomised controlled trials have addressed this topic in patients with neurological disorders, thus pointing to the weak internal validity of the current clinical evidence. Future studies should propose easier solutions to be applied in unsupervised settings without requiring technical expertise (e.g., issues related to data storage, access platforms and software/app usage). As a possible solution, machine-learning algorithms, including those using artificial neural networks (deep learning algorithms) [221], would be suitable tools for the automatic storage, interpretation and management of healthcare data [222,223,224]. Indeed, by learning from massive amounts of longitudinal data, machine-learning systems could lighten the burden of technical expertise and improve clinical decision making through a tailored approach. Another relevant point concerns some ethical issues, such as the security of the overwhelming amount of healthcare sensitive data derived from the use of wireless sensors, possibly leading to the generation of discriminatory profiles, manipulative marketing or data breaches [225]. Accordingly, limiting the wireless transmission to a small number of selected data (e.g., fall episodes) would help to preserve the confidentiality of a large amount of recorded information in case of privacy violation. Using proper encryption technology and increasing the users’ awareness of privacy rights would help to address ethical issues. Nonetheless, strict regulations for data management should also be adopted to guarantee users’ confidentiality and integrity [226]. The use of inertial sensors included in smartphones would address the issue of the cost and availability of wearable sensors [227]. 

## 9. Conclusions

Over the last 15 years, wearable devices have been largely used for the assessment of balance in patients affected by neurological disorders, providing valuable data compared with standard laboratory instrumentation. Indeed, a great experience in the use of wireless sensors for balance evaluation has been achieved in the laboratory setting. Conversely, much still needs to be done for the technological migration of wearable devices from the laboratory to the domestic unsupervised environment. This migration would open several valuable prospects, including teleneurology and telerehabilitation approaches.

## Figures and Tables

**Table 1:** Balance impairment in neurological disorders.

	Disease Definition	Nervous Structures Involved	Pathophysiological Mechanisms	Main Clinical Consequence
Alzheimer’s disease	Neurodegenerative dementia associated with progressive cognitive and functional dysfunction [27]	Cerebral cortex and subcortical structures, prominently involving nucleus accumbens and putamen [28]	Cognitive impairment, abnormal sensorimotor function and vision, peripheral sensory loss, muscle weakness [29,30,31,32]	Hallucinations, inattention, abnormal sensory reweighting
Parkinson’s disease	Neurodegenerative movement disorder associated with progressive motor and cognitive dysfunction [33]	Basal ganglia, locus coeruleus and pedunculopontine nucleus [34]	Impaired scaling of postural responses [35], abnormal central proprioceptive-motor integration [36], reduced kinaesthesia [37], axial rigidity [38], cognitive dysfunction [39]	Postural instability, disrupted trunk-legs coordination, freezing of gait
Multiple sclerosis	Acquired demyelinating disease of the central nervous system [40]	Cortico-spinal tract, cerebellum, proprioceptive pathways, vestibular system, brainstem structures for eye movement control [41]	Abnormal sensorimotor, visual, cerebellar, vestibular and cognitive functions [41], muscle weakness and spasticity [42]	Abnormal coordination and sensory reweighting, reduced attentional resources, strength impairment
Huntington’s disease	Neurodegenerative disease with autosomal dominant pattern of inheritance [43], associated with cognitive and motor impairment, psychiatric disorders and involuntary movements (chorea) [44]	Basal ganglia, prominently interesting caudate and putamen [45]	Involuntary movements, trunk muscles weakness, hip flexor tightness, impairment in visual and vestibular integration, ocular pursuit movements and proprioception [46]	Chorea, abnormal sensory reweighting, increased stride variability
Cerebellar ataxia	Acquired or hereditary, as well as acute or progressive, disorder associated with dysfunction of cerebellum and/or its connections [47]	Cerebellum (primarily vermis and anterior lobe) and/or its connections, including spinocerebellar tracts [47]	Impaired coordination of movements	Axial motor impairment and asynergic movement
Stroke	Acute neurologic syndrome due to the interruption of blood supply to a part of the central nervous system by an ischemic or haemorrhagic vascular injury [48]	Cortico-spinal tract, cerebellum, proprioceptive pathways, vestibular system and brainstem structures [49]	Somatosensory and motor dysfunction [50,51], spasticity [52], visual and perceptual disorders [53,54], including impaired perception of upright body position, cognitive impairment [55]	Hemispatial neglect, strength impairment, abnormal coordination, sensory reweighting
Traumatic brain injury	Acute blunt head traumas or acceleration forces to the head [56]	Vestibular nuclei, cerebellar peduncles, medial lemniscus, dentato-rubro-thalamic and cortico-reticular pathways [57]	Impairment in cognitive and motor functionality [58]	Dizziness, visual-spatial deficits and inattention
Neuropathies	Acute or progressive disorders of the peripheral nervous system, associate with the disruption of nerve action potentials transmission [59]	Peripheral nervous system (nerves)	Sensory and/or motor impairment [59], retinopathy, vestibular and muscle impairment [60], sensory ataxia	Proprioception and strength impairment
Vestibular syndromes	Acute or chronic disorders of the inner-ear balance organs and/or their nervous structures [61] (e.g., Meniere’s disease, benign positional vertigo, bilateral vestibular loss, vestibular neuritis, posterior circulation strokes)	Vestibular system (i.e., inner-ear balance organs, vestibular nerve and central nuclei)	Abnormal spatial orientation and motion perception [62], ataxia, eye movement abnormalities [61]	Dizziness and vertigo

**Table 2:** Standardised clinical tests and scales for balance assessment.

Clinical Test or Scale	Aim of the Test/Scale	Procedures	Outcome Measures
Romberg test [64]	Postural ability and pathophysiological mechanisms	The subject stands with feet close together, arms by the side, and with eyes open, and then closes eyes while maintaining the same position (removal of vision possibly compensatory proprioceptive deficits)	Unbalance and fall
Pull test [65]	Postural ability	The subject undergoes a sudden body displacement by a quick and forceful pull on the shoulders during upright stance	Number of backward steps or falling (qualitative)
Tandem gait test [66]	Postural ability	The subject walks a straight line while touching the heel of one foot to the toe of the other (narrowed base of support)	Unbalance, falls or need to enlarge the base of support
One-leg stance test [67]	Postural ability	The subject stands unassisted on one leg with opened eyes and arms on the hips as long as possible	Time of performance in seconds
Timed up and go test [68]	Gait and postural ability	The subject sits on a chair, stands up, walks 3 m, turns around, walks back and sits down	Time of performance in seconds
Tinetti balance and mobility scale - Performance-oriented mobility assessment [69]	Gait and postural ability	The subject performs postural and walking motor tasks reflecting common daily activities, such as rising from a chair, maintaining upright stance after a nudge, walking and turning (total 24 items consisting of 14 balance items and 10 gait items)	Total score (sum of gait and balance scores) by using a 2/3-point ordinal scale for each item
Functional reach test [70]	Postural ability	The subject reaches as far forward as he can with arms at 90° flexion, keeping feet on the floor	Maximum distance (cm) that the subject can reach forward beyond arm’s length
Berg balance scale [71]	Postural ability	The subject performs functional activities reflecting different components of postural control, such as reaching, bending, transferring and standing (total 14 items)	Total score by using a 5-point ordinal scale for each item
Activities of balance confidence scale [72]	Postural ability	The subject performs a self-report questionnaire on subjective impact of balance dysfunction on 16 daily activities, such as walking in different environmental and postural conditions (total 16 items)	Average score in percentage (each item rated from 0% to 100% of balance confidence)
Physiological profile assessment [73]	Pathophysiological mechanisms	The subject performs different sensorimotor tasks to assess vision (e.g., dual contrast visual acuity chart), lower limb sensation (e.g., tests of proprioception), legs strength, step reaction times, vestibular function (e.g., visual field dependence) and postural sway	Falls risk assessment based on the scores of sensorimotor tasks
Balance evaluation systems test [74]	Pathophysiological mechanisms	The subject performs several motor tasks reflecting different systems underlying balance control (e.g., stance on a firm or foam surface, stepping over obstacles, alternate stair touching); (total 36 items categorised into 6 underlying systems: "Biomechanical Constraints," "Stability Limits/Verticality," "Anticipatory Postural Adjustments," “Postural Responses,” “Sensory Orientation” and “Stability in Gait”)	Total score in percentage referring to the partial score of systems that involve a 4-point ordinal scale for each item

**Table 3:** Main biomechanical parameters for balance assessment through traditional and wearable instrumentation.

Name	Meaning	Static	Dynamic
RANGE	Range of acceleration/displacement in the AP, ML, and V direction. Impaired motor strategies report high values of Range Index	[114,117,118]	[111,119]
STD	Standard deviation of reference body landmarks. It is an index of average amplitude of body displacements.		[102,104,120,121]
DIST	Mean distance from the centre of acceleration/displacement trajectory. It is an index of desertion. In static evaluation, high values indicate poor motor control.	[114,117,122,123]	
RMS	Root mean square of the acceleration/displacement in AP, ML, and V direction. High values represent larger dispersion and poor motor control.	[114,117,122,123,124,125,126,127]	[128]
MEAN	Average acceleration/velocity/displacement in the AP, ML, V direction. High values represent unstable postural adjustments and poor motor control.	[118,122,127]	
PATH	Total length of the acceleration/displacement in static condition larger values represent poor motor control.	[114,117]	[26,102,128]
MV	Mean velocity. It is the first derivative of the acceleration signal in the AP, ML and V direction. Impaired motor strategies report High values of Mean Velocity Index.	[114,117]	
AREA	Total area that encapsulates the total sway path in AP and ML directions. In a static condition, higher values represent poor motor control.	[114,117,118,123,127]	
EA95	95% ellipse sway area. It is the ellipse area that encapsulates the 95% of the sway path in the AP and ML direction. High values represent poor motor control.	[114,117,126,127]	
JERK	Time derivative of the acceleration signal. It represents the range of changes in the acceleration signal. High values represent accelerating and decelerating pattern attesting more unstable condition and poor motor control.	[114,117,118,122,125]	
Cross-correlation	Cross-correlation between displacements of two body points. It is an index of coupling between the motion behaviour of two body segments or between the movable platform and the human body		[102,104,120,129]
PWR	Total power of the power spectrum of the acceleration signal.	[114,123]	[102]
F95 or F50	Frequency below which is present the 95% or 50% of the total power. High values indicate a larger amount of postural adjustments and poor motor control.	[114,118]	
CF	Centroidal frequency of the signal in the AP, ML and V direction. It is the frequency at which the power is balanced, i.e., the total power above this frequency is equal to the one below. Poor motor control is identified by low values of CF.	[114,117,122]	
FD	Frequency dispersion. It is a measure of the variability of the frequencies of the power spectral density. Values close to zero indicate pure sinusoidal patterns of the signal and a more stable motor control.	[114,117,118,122]	
Entropy	It is the power spectrum entropy of the signal. It is an index of movement smoothness and the inability to regulate postural fluctuations.	[127,130]	
Magnitude	It the area below the EMG curve over a specific range of time, starting from the onset of the perturbation. Mostly this index of muscular intensity is computed during the early response (0–200 ms), the intermediate response (201–400 ms) and the late response (401–600 ms). Impaired postural strategies report lower values of muscle activation.		[111,119]
Onset latency	Time delay between onset of perturbation and muscle activation. It represents how fast a muscle reacts after a perturbation. Impaired balancing strategies report high values of onset latency.		[86,90,111,119]
Time to peak	Time between the onset of perturbation and the maximum activation of the muscle or the maximum peak of joint angle. It indicates how quickly a muscle/joint reaches its maximal value. In dynamic evaluation, lower values indicate high capability in counteracting perturbation.		[86,90,111,119,129,131]
Coactivation	It is the ratio between the magnitude of the agonist and antagonist muscles activity. Impaired postural strategies present an increased coactivation of agonist-antagonist muscles.		[86,90]
Peak angle	Peak of the angular displacement of two adjacent body segment.		[86,129,131]
APAs–CPAs	Anticipatory and compensatory postural adjustments. EMG activity and principal component analysis are estimated over four-time windows in relation to perturbation onset, i.e., APA1 (from −250 ms to −100 ms); APA2 (from −100 ms to +50 ms); CPA1 (from +50 ms to +200 ms); CPA2 (from 200 ms to +350 ms). Impaired motor control reports smaller and delayed APAs during unexpected perturbation.		[95,105,106,109]

AP: antero-posterior; APA: anticipatory postural adjustment; CPA: compensatory postural adjustment; EMG: electromyography; ML: medio-lateral; V: vertical.

**Table 4:** Strengths, limitations and challenges of wireless sensors currently available for balance assessment.

Wireless Sensor	Strengths	Limitations	Challenges
IMU	Low cost and high accuracy	Possible magnetic interferences, errors of misalignment, orthogonality and offset and energy consumption	New algorithms for position and orientation correction
sEMG	Noninvasive analysis and unobtrusiveness	Crosstalk due to adjacent muscles, skin-electrode interface noise and electrode positioning	New implantable EMG sensors and dry electrodes composed of conductive fabric
Pressure	Outdoor measurements and easy integrability	Low comfortability during gait, limited sensitive area and high cost	New capacitive sensors composed of fabric

**IMU**: Inertial Measurement Unit; **sEMG**: surface electromyography

**Table 5:** Sensor-based balance evaluation in neurological disorders.

Disease and Number of Studies	Studies with a Control Group	Type and Main Locations of Sensors	Other Measurements	Main Experimental Setups	Main Postural Measures	Main Findings	Clinical-Behavioural Correlations
Alzheimer’s disease N = 3 [32,202,203]	N = 3 [32,202,203]	1 to 5 IMUs on trunk, waist, legs and thighs	Not performed	Upright stance with open or closed eyes, different BOS amplitudes and surfaces (e.g., firm and foam), as well as during virtual perturbations	Pitch and roll angles; COM displacement; sway velocity, area and path; RMS acceleration	Lower minimal roll angle, larger COM displacement, higher sway area and RMS acceleration in AD than HS	Not significant or not performed
Parkinson’s disease N = 17 [114,122,124,125,156,157,158,159,160,161,162,163,164,165,166,167,168]	N = 16 [114,122,124,125,156,157,158,159,160,161,162,163,164,165,166,168]	1 to 8 IMUs on trunk, waist, wrists, thighs, shanks and feet; 10 to 22 sEMG on lower limb muscles, lumbar erector spinae, thoracic erector spinae and rectus abdominis	Force plate (COP measures) and infrared optical system	Gait initiation; upright stance with open or closed eyes, different BOS amplitudes and surfaces (e.g., firm and foam), under and not under cognitive load; SOT; ISAW; self-triggered and external postural perturbations; OLS	IMUs: APAs; mean velocity; RMS acceleration; jerkiness; peak-to-peak sway; 95% ellipse area; strategy index. sEMG: amount of variance accounted for; synergy index; ASAs; modulation index	Correlation between inertial, COP and optical measures; hypometric APAs, higher mean velocity, acceleration size and jerkiness, larger peak-to peak sway and 95% ellipse area, predominant ankle strategy; lower VAF and synergy index, reduced ASAs and muscle modulation in PD than HS	Acceleration changes correlated with PIGD and UPDRS-III scores, strategy index with ABC scores, muscle modulation with postural ability and disease severity in PD
Multiple sclerosis N = 11 [118,146,169,170,171,172,173,174,175,176,177]	N = 10 [118,146,169,170,171,172,173,174,175,177]	1 to 6 IMUs on trunk, waist, wrists, thighs, shanks and feet	Force plate (COP measures) and infrared optical system	Upright stance with open or closed eyes and different surfaces (e.g., firm and foam); walking tasks (e.g., TUG, timed 25-foot walk, 6-minute walk test); external perturbations (e.g., push and release test, backward perturbation)	RMS acceleration; mean velocity; sway jerk, path length, area; F95%; time to reach stability; coherence of acceleration between trunk and legs	Correlation between inertial and COP measures; larger sway acceleration amplitude, angular trunk range of motion in roll and yaw axes, sway path length and area, reduced ML sway jerk, higher F95%, longer time to reach stability and lower acceleration coherence between trunk and legs in MS than HS	Sway acceleration correlated with ABC and MSWS12 scores; RMS acceleration, displacement, mean frequency and time to reach stability correlated with EDSS scores
Huntington’s disease N = 2 [46,204]	N = 2 [46,204]	1 to 2 IMUs on trunk and waist	Not performed	Upright stance with open or closed eyes and different BOS amplitudes; sitting, standing and walking	RMS acceleration; total, peak and mean angular excursion	Higher RMS acceleration; larger peak and total excursions in HD than HS	Not significant or not performed
Cerebellar ataxia N = 7 [130,190,191,192,193,194,195]	N = 7 [130,190,191,192,193,194,195]	1 to 6 IMUs on trunk, waist, wrists, ankles and feet	Force plate (COP measures)	Upright stance with open or closed eyes and different surfaces (e.g., firm and foam); walking tasks and external perturbations (e.g., retropulsion test)	Trunk angular displacement and velocity, sway path length, area of the convex hull, convex polyhedron volume, entropy, 95% of the ellipse sway area	Correlation between inertial and COP measures; larger trunk angular displacement and velocity, sway path length, area of the convex hull, convex polyhedron volume, entropy and 95% of the ellipse sway area in CA than HS	Inertial measures (e.g., trunk angular displacement and velocity) correlated with ICARS scores, Tinetti’s Mobility Index and SARA scores
Stroke N = 8 [52,178,179,180,181,182,183,184]	N = 5 [179,181,182,183,184]	1 to 5 IMUs on head, trunk, waist and shins	Force plate (COP measures)	Upright stance with open or closed eyes and different BOS amplitudes; walking tasks; functional reach test; Fukuda stepping test; OLS	Body displacement (time, velocity, acceleration); RMS acceleration	Higher maximum and minimum acceleration, LL trunk acceleration, angular velocity in ST than HS	Gyroscope data negatively correlated with Berg balance scale scores
Traumatic brain injury N = 7 [123,126,185,186,187,188,189]	N = 6 [123,126,185,186,188,189]	1 IMU on waist	Force plate (COP measures)	Upright stance with open or closed eyes, different BOS amplitudes and surfaces (e.g., firm and foam); standard and modified balance error scoring system	RMS acceleration; sway amplitude, velocity, variability and frequency; ellipse and total sway area; 95% ellipsoid sway volume	Higher RMS, total power, mean distance, acceleration range, path length, ellipse and total sway area, 95% ellipsoid sway volume and area in TBI than HS	Self-reported symptoms (e.g., dizziness, headache) correlated with sway path length and postural sway area
Neuropathies N = 3 [199,200,201]	N = 3 [199,200,201]	1 to 2 IMUs on waist and shin	Force plate (COP measures)	Upright stance with open or closed eyes, different BOS amplitudes and surfaces (e.g., firm and foam)	RMS acceleration; range of acceleration; peak velocity; body sway area	Correlation between inertial and COP measures; higher RMS acceleration, acceleration range, and peak velocity; larger body sway area in NP than HS	Vibration perception threshold negatively correlated with postural control
Vestibular syndromes N = 4 [196,197,198,199]	N = 4 [196,197,198,199]	1 to 4 IMUs on head, trunk, waist and legs	Not performed	Upright stance with open or closed eyes, different BOS amplitudes and surfaces (e.g., firm and foam); walking tasks; shortened functional mobility test	Range of acceleration; peak velocity; RMS acceleration; mean power frequency; quotient of Romberg for inertial measures	Higher range of acceleration, peak velocity, RMS acceleration and quotient of Romberg for some inertial measures; smaller mean power frequency in VS than HS	Not significant or not performed

ABC: Activities-Specific Balance Confidence Scale; AD: patients with Alzheimer’s disease; ASA: Anticipatory Synergy Adjustment; APA: anticipatory postural adjustment; BOS: base of support; CA: patients with cerebellar ataxia; COM: centre of mass; COP: centre of pressure; EDSS: Expanded Disability Status Scale; F95%: frequency comprising 95% of the signal; HD: patients with Huntington’s disease; HS: healthy subjects; ICARS: International Cooperative Ataxia Rating Scale; IMU: Inertial Measurement Unit; ISAW: Instrumented Stand and Walk Test; LL: latero-lateral; ML: medio-lateral; MS: patients with multiple sclerosis; MSWS12: 12-Item Multiple Sclerosis Walking Scale; N: number; NP: patients with neuropathies; OLS: one-leg stance; PIGD: Postural Instability/Gait Difficulty score; PD: patients with Parkinson’s disease; RMS: root mean square; SARA: Scale for the Assessment and Rating of Ataxia; sEMG: surface electromyographic sensors; SOT: Sensory Organisation Test; ST: patients with previous stroke; TBI: patients with previous traumatic brain injury; TUG: Timed-Up and Go test; UPDRS-III: Unified Parkinson’s Disease Rating Scale—part III; VAF: variance accounted for; VS: patients with vestibular syndrome.

## Supplementary Materials

The following are available online at https://www.mdpi.com/1424-8220/20/11/3247/s1, Table S1: previous reviews on balance and fall risk assessment through wireless sensors and Table S2: sensor-based balance evaluation in neurological disorders. References [32,46,52,114,115,118,122,123,124,125,126,130,146,147,156,157,158,159,160,161,162,163,164,165,166,167,168,169,170,171,172,173,174,175,176,177,178,179,180,181,182,183,184,185,186,187,188,189,190,191,192,193,194,195,196,197,198,199,200,201,202,203,204,211,216] are cited in the supplementary materials.

## Abbreviations

APA	Anticipatory postural adjustment
AT	Adaptation Test
BOS	Base of support
IMU	Inertial measurements unit
COM	Centre of mass
COP	Centre of pressure
MCT	Motor Control Test
sEMG	Surface electromyography
SOT	Sensory Organisation Test
