# Supplementary material for: Fifteen Years of Wireless Sensors for Balance Assessment in Neurological Disorders
Source: Sensors (Basel). 2020 Jun 7;20(11):3247. doi: 10.3390/s20113247 (PMC7308812; doi:10.3390/s20113247)
Supplement: Supplementary file 1 [file sensors-20-03247-s001.pdf]

Table S1. Previous reviews on balance and fall risk assessment through wireless sensors.

Reference	Year	Subjects	Topic	Study objectives	Studies included
Diaz et al. [1]	2020	OA, PD, MS, AD	Use of wearable technologies in walking, balance and range of motion analysis	Design issues, outcome measures, biofeedback, measure of validity, machine learning approaches	56
Ghislieri et al. [2]	2019	YA, OA, SRC, PD, MS, AS, TBI, DM, CA, ST, and HM	Novel posturographic paradigm for the analysis of the human postural sway through inertial sensors	Sensors types and placement, test protocols, balance measures, measure of validity	47
Pinho et al. [3]	2019	YA and OA	Mobile devices in the assessment of postural balance of healthy subjects	Balance protocols, sensors type and positions, mobile apps, outcome measures	9
Pang et al. [4]	2019	YA, OA, PD, and ST	Assessment of "near falls", such as slips, trips, stumbles, missteps, incorrect weight transfer, or temporary loss of balance, using wearable devices	Type of falls, sensors placement, algorithms developed, measure of validity	9
Moral-Munoz et al. [5]	2018	N/A	Smartphone applications for the balance assessment	App quality scores and app subjective quality score	N/A
Sun et al. [6]	2018	MS	Sensing technologies in the assessment of mobility and balance impairments	functional assessment protocols, outcome measures, measure of validity and reliability	33
Sun et al. [7]	2018	OA	Sensing technology in providing objective fall risk assessment in older adults	Sensor placement, Test Protocol, outcome measures, measure of validity	22
Gordt et al. [8]	2017	PD, ST, PN, and OA	Meta-analysis of randomized controlled trials on wearable sensors technologies for the assessment of balance and gait training	Sensors type and placement, type of feedback, training modalities, outcome measures	8
Roeing et al. [9]	2017	YA, OA, PD, and VD	Mobile health apps for testing balance as a fall risk factor	Clinical tests, measure of balance, measure of validity, measure of reliability	13
Godinho et al. [10]	2016	PD	Wearable, non-wearable and hybrid devices used for the clinical assessment of PD	Monitoring technologies, measure of validity, motor disability	73

Ma et al. [11]	2016	YA, ST, OA, PD, DM, SP, AM	Effect of biofeedback systems, with wearable inertial motion sensors and force sensors, on balance performance	Sensors type, sensors location, biofeedback type, outcome measure	17
Hubble et al. [12]	2015	PD	Wearable sensors in the estimation of standing balance and walking stability in PD	Sensors type, sensors placement, measure of balance and stability, test modality	26
Maetzler et al. [13]	2013	PD	Recent innovations in clinical management by using wearables	Sensors placement, motor disability, non-motor disability, measure of balance, devices used	32
Howcroft et al. [14]	2013	OA	Fall risk assessment by using wearable inertial-sensor-based systems	Sensor placement, derived parameters used to assess fall risk, fall risk classification method, and fall risk classification model outcomes	40

AD: Alzheimer's Disease; AM: Amputees; AS: Ankle Sprain; CA: Cerebellar Ataxia; DM: Diabetes Mellitus; HM: Haemophilia; MS: Multiple Sclerosis; N/A: Not Applicable; OA: Older Adults; PD: Parkinson's Disease; PN: Peripheral Neuropathy; SP: Subjects with Paraplegia; SRC: patients with Sport-Related Concussions; ST: Stroke; TBI: Traumatic Brain Injury; VD: patients with Vestibular Dysfunction; YA: Young Adults

Table S2. Sensor-based balance evaluation in neurological disorders.

Author/Year	Participants (mean age ± SD)	Type and location of wearable sensors	Other measurement	Experimental setup	Main postural measures	Main findings	Clinical-behavioural correlations
**Alzheimer's disease**							
Gago et al. (2014) [15]	9 AD non-fallers (73.56 ± 9) 11 AD fallers (77.64 ± 5) 16 HS (72.31 ± 7)	5 IMU on trunk, legs and thighs	Not performed	Romberg test on flat and inclined surfaces	Pitch and roll angles; total and maximal COM displacement; maximal linear velocity	Larger COM displacement in AD fallers than HS when eyes closed on flat surface; lower minimal roll angle in AD then HS when eyes closed on frontward platform	Not significant correlations
Hsu et al. (2014) [16]	21 AD (61.48 ± 5) 50 HS (59.86 ± 5)	1 inertial sensor (accelerometer + uniaxial gyroscope + biaxial	Not performed	Side-by-side, tandem and one-leg upright stance,	Sway speed in AP and ML directions	Greater ML speed in tandem stance with eyes closed and in one-leg stance	Not performed

		gyroscope) on waist		with eyes open and eyes closed			
Gago et al. (2016) [17]	9 AD non-fallers (75)<br>11 AD fallers (76)<br>21 HS (71)	1 IMU on the back (55% of patient's height above the ground)	Not performed	Upright stance with virtual unpredictable visual displacements and falling	COM displacement; sway area and path; RMS acceleration	Higher range of acceleration on z-axis, mean and RMS acceleration and average acceleration magnitude in AD fallers than HS	Not performed
				**Parkinson's disease**			
Mancini et al. (2009) [18]	11 PD (60.3 ± 0.7)<br>12 HS (not specified; age-matched)	3 inertial sensors (accelerometer + gyroscope) on C7, L5 and right thigh	Force plate (COP measures)	Taking two steps	APAs duration; peak AP and ML acceleration; time-to-peak angular velocity; thigh range of motion	Linear correlation between COP and inertial measures; smaller peak ML acceleration and hypometric APAs in ML direction	Not performed
Mancini et al. (2011) [19]	13 PD (60.4 ± 8)<br>12 HS (60.2 ± 8)	1 inertial sensor (accelerometer + gyroscope) on L5	Force plate (COP measures)	Upright stance with eyes open, eyes closed and eyes closed during a cognitive task	RMS of acceleration; mean velocity; F95%; frequency dispersion; jerk	Larger RMS, mean velocity and jerk in eyes open condition	Not significant correlations
Mancini et al. (2012) [20]	17 PD (67.1 ± 7)<br>17 HS (67.9 ± 6)	1 inertial sensor (accelerometer) on L5	Force plate (COP measures)	Upright stance with eyes open	Jerk; Time-domain (e.g. RMS, mean velocity, sway area) and frequency-domain (e.g. F95%, frequency dispersion) measures	Similar sensitivity of COP and inertial measures;<br>larger size and jerkiness of accelerations;<br>high test-retest reliability of jerk and time-domain measures	Acceleration measures correlated with postural impairment (e.g. PIGD scores)

Table S2. Sensor-based balance evaluation in neurological disorders.

Author/Year	Participants (mean age ± SD)	Type and location of wearable sensors	Other measurement	Experimental setup	Main postural measures	Main findings	Clinical-behavioural correlations
**Parkinson's disease**							
Maetzler et al. (2012) [21]	12 PD (61.5 ± 2)<br>14 HS (63.9 ± 2)	1 inertial sensor on L3-L4	Not performed	Upright semitandem stance with eyes open and closed, on firm and foam surfaces	RMS of AP and ML acceleration; mean velocity; F95%; jerk	Comparable values	Not performed
Baston et al. (2014) [22]	4 PD (62 ± 6)<br>7 HS (68 ± 7)	2 inertial sensors on L5 and right shank	Not performed	SOT	Covariance index between the trunk and shank; strategy index (hip or ankle strategy); RMS of AP acceleration	Larger time in in-phase pattern reflecting predominant adoption of ankle strategy; poor change of postural strategies; similar RMS values	Not performed
Curtze et al. (2016) [23]	104 PD (66.5 ± 6)<br>64 HS (65.4 ± 6)	6 inertial sensors on wrists, sternum, L5 and ankles	Not performed	Instrumented Stand and Walk test OFF and ON state of therapy	RMS of AP and ML acceleration, mean velocity, centroidal frequency, frequency dispersion, jerk; APA duration, latency and peak AP and ML	Larger RMS, mean velocity, centroidal frequency and jerk; Dopaminergic therapy increased RMS and mean velocity especially in dyskinetic PD; larger APAs in ON than OFF state of therapy	Not performed
Mancini et al. (2016) [24]	10 PD (67.2 ± 5)<br>12 HS (68 ± 6)	3 IMUs on L5 and shanks	Force plate (COP measures); infrared optical system	Gait initiation trials	APAs AP and ML peak; APA duration	Linear correlation between inertial, COP and optical measures; smaller APAs measures	Not significant correlations

Baston et al. (2016) [25]	70 PD (67 ± 6)<br>21 HS (67 ± 6)	2 inertial sensors on L5 and right shank	Not performed	Upright stance OFF and ON state of therapy	Strategy index (hip or ankle strategy); RMS of AP acceleration	Larger RMS of AP acceleration;<br>more ankle strategy and larger RMS of AP acceleration during OFF than ON state of therapy	Strategy index and RMS values correlated with motor impairment (e.g. UPDRS-III and PIGD scores); strategy index correlated with balance confidence (e.g. ABC scores)

Table S2. Sensor-based balance evaluation in neurological disorders.

Author/Year	Participants (mean age ± SD)	Type and location of wearable sensors	Other measurement	Experimental setup	Main postural measures	Main findings	Clinical-behavioural correlations
				**Parkinson's disease**			
Falaki et al. (2016) [26]	11 PD (69.4 ± 6.3)<br>11 HS (65.3 ± 8.1)	sEMG sensors on 10 lower limb muscles, lumbar erector spinae, thoracic erector spinae and rectus abdominis (right-sided)	Force plate (COP measures)	Quiet standing, voluntary sway, fast-sway and load release (self-triggered postural perturbations)	Initiation of APAs; amount of variance 4 muscle modes account for; synergy index; anticipatory synergy adjustments	Similar APAs; lower amount of variance 4 muscle modes account for; lower synergy index during steady state; reduced anticipatory synergy adjustments	Not performed
Falaki et al. (2017) [27]	10 PD (69.4 ± 6.3)<br>No HS	sEMG sensors on 10 lower limb muscles, lumbar erector spinae,	Force plate (COP measures)	Quiet standing; voluntary sway, load release, fast-body motion (self-triggered	Initiation of APAs; variance in muscle activation; synergy index; anticipatory	Similar APAs OFF and ON state of therapy; larger indices of synergies (e.g.	Not performed

		thoracic erector spinae and rectus abdominis (right-sided)		postural perturbations) OFF and ON state of therapy	synergy adjustments	variance in muscle activation) and anticipatory synergy adjustments ON than OFF state of therapy	
de Souza Fortaleza et al. (2017) [28]	30 PD without FOG (68.6 ± 8)<br>26 PD with FOG (69.2 ± 8)<br>15 HS (not specified; age-matched)	8 inertial sensors on feet, shanks, wrists, chest and L5	Not performed	Instrumented Stand and Walk test standard and during a cognitive task	RMS of AP and ML acceleration; AP and ML jerk; APA duration and AP and ML peak amplitude	Larger RMS of AP acceleration in PD with FOG than PD without FOG	Not performed
Ozinga et al. (2017) [29]	14 PD (63 ± 8)<br>14 HS (65 ± 9)	1 inertial sensor (accelerometer within a tablet computer – iPad) on the waist	Force plate (COP measures)	SOT	Peak-to-peak AP and ML COM acceleration; normalized path length; RMS of AP and ML acceleration; 95% ellipse area; equilibrium score	Linear correlation between COP and COM measures; larger peak-to-peak, normalized path length, RMS and 95% Ellipse Area in AP and ML directions during SOT5 and SOT6	Not performed
Ozinga et al. (2017) [30]	27 PD (62.9 ± 9)<br>27 HS (not specified; age-matched)	1 inertial sensor (accelerometer + gyroscope within a tablet computer – iPad) on waist	Not performed	Double-leg and tandem upright stance, with eyes open and closed, on firm and foam surfaces	Cleveland clinic-postural stability index (reflecting peak-to-peak sway)	Greater peak-to-peak measures in all SOT conditions	Cleveland clinic-postural stability index correlated with postural impairment (e.g. PIGD scores)
Bonora et al. (2017) [31]	33 PD without FOG (67.5 ± 8)<br>25 PD with FOG (67.0 ± 6)<br>32 HS (69.4 ± 7)	3 IMUs on L4-L5 and tibias	Not performed	OLS	ML-peak trunk acceleration (reflecting APAs); RMS of AP and ML acceleration	Lower ML-peak in PD than HS; Larger RMS of AP and ML acceleration (OLS on the most affected side)	ML-peak acceleration correlated with motor impairment (e.g. UPDRS-III)

Table S2. Sensor-based balance evaluation in neurological disorders.

**Author/Year**	**Participants (mean age ± SD)**	**Type and location of wearable sensors**	**Other measureme nt**	**Experimental setup**	**Main postural measures**	**Main findings**	**Clinical-behavioural correlations**
**Parkinson's disease**							

Bonora et al. (2017) [32]	10 PD (67.2 ± 5)<br>12 HS (68 ± 5)	3 IMUs on L5 and shins	Force plate (COP measures)	3 gait initiation trials	Amplitude of ML trunk acceleration (APAs); ML angular velocity	COP and COM measures significantly correlated; smaller ML trunk acceleration; longer unloading phase	Not performed
Chen et al. (2018) [33]	23 PD (66.2 ± 8)<br>23 HS (64.2 ± 7)	1 inertial sensor (accelerometer) on L4-L5	Not performed	Upright stance with eyes open and eyes closed, as well as eyes open and eyes closed during a cognitive task	RMS of AP and ML acceleration; AP and ML jerk	Larger RMS and jerk values with and without eyes open during a cognitive task	Not performed
Lang et al. (2019) [34]	31 PD (68 ± 9)<br>13 HS (65 ± 9)	sEMG sensors on 11 lower limb muscles (bilaterally)	Infrared optical system	Multidirectional support surface translation perturbations	Modulation index (considering medium- and long-latency automatic postural responses) reflecting the ability to appropriately inhibit muscles according to the balance task	Lower muscle modulation across perturbation directions regardless of PD phenotype	Association of PD, PD severity, balance ability and FAB scores with muscle modulation
**Multiple sclerosis**							
Spain et al. (2012) [35]	31 MS (39.8)<br>28 HS (37.4)	6 inertial sensors (accelerometer + gyroscope) on shins, wrists, sternum and L5	Not performed	Upright stance with eyes open and eyes closed; timed 25-foot walk; timed up-and-go test	RMS of AP and ML acceleration; mean sway velocity; sway frequency; sway jerk; trunk range of motion	Larger sway acceleration amplitude and lower ML normalized jerk in upright stance with eyes closed; greater increase of sway	Sway acceleration amplitude negatively correlated with balance confidence and perceived

						acceleration amplitude with closure of eyes; larger angular trunk range of motion in roll and yaw axes during walking tasks	walking abilities (e.g. ABC and MSWS12 scores)
Spain et al. (2014) [36]	27 MS (41)<br>18 HS (34)	6 inertial sensors (accelerometer + gyroscope) on shins, wrists, sternum and L5	Not performed	Upright stance with eyes open and eyes closed; timed 25-foot walk	Sway acceleration amplitude; sway jerk; sway area; trunk yaw range of motion	Larger sway area, trunk yaw range of motion; reduced normalized ML jerk	Not performed

Table S2. Sensor-based balance evaluation in neurological disorders.

Author/Year	Participants (mean age ± SD)	Type and location of wearable sensors	Other measurement	Experimental setup	Main postural measures	Main findings	Clinical-behavioural correlations
			**Multiple sclerosis**				
Solomon et al. (2015) [37]	20 MS (40)<br>20 HS (not specified; age-matched)	6 inertial sensors (accelerometer + gyroscope + magnetometer) on sternum, lumbar region, wrists and ankles	Not performed	Upright stance with eyes open and eyes closed, on foam surface	46 measures of velocity, acceleration, jerk and spectral power of sway	Larger sway path length and range of sway acceleration amplitude in ML direction (independent predictors to differentiate MS from HS)	Range of sway acceleration amplitude correlated with balance confidence and perceived walking abilities (ABC and MSWS-12 scores)
Craig et al. (2017) [38]	15 MS (48.2 ± 9<br>15 HS (47.8 ± 9)	6 inertial sensors (accelerometer + gyroscope +	Not performed	Upright stance with eyes open; timed up-and-go test	Sway jerk, area, RMS, mean velocity, 95%	Good to excellent test-retest reliability of considered	RMS, range of displacement and mean frequency

		magnetometer) on sternum, L5, wrists and ankles			power frequency, frequency dispersion, trunk range of motion and velocity	measures (except frequency dispersion)	correlated with disability (e.g. EDSS scores)
El-Gohary et al. (2017) [39]	52 MS (49.5 ± 10) 21 HS (49.9 ± 12)	3 inertial sensors (accelerometer + gyroscope) on lumbar region and feet	Force plate (COP measures); infrared optical system	Push and release test	Latency of Postural Response; time of first heel strike; time to reach stability; number of steps; step length	Measures by means of inertial sensors correlated with laboratory reference measures; longer time and more steps to reach stability	Time to Reach Stability and step latency correlated with disability (e.g. EDSS scores)
Witchel et al. (2018) [40]	17 MS (53.06 ± 11) 23 HS (46.13 ± 11)	3 inertial sensors (accelerometer + gyroscope + magnetometer) on L3 and thighs	Not performed	Timed-up-and-go test (sit-to-stand and stand-to-sit transitions)	Angular velocity features (area under the curve, absolute peak and absolute mean); normalized mean absolute jerk; speed arc length	Lower thigh pitch angular velocity in sit-to-stand transition; larger roll peak in stand-to-sit transition	Not performed
Huisinga et al. (2018) [41]	36 MS (45.6 ± 12) 20 HS (41.8 ± 11)	6 inertial sensors (accelerometer + gyroscope + magnetometer) on sternum, L5, wrists and ankles	Force plate (COP measures)	Upright stance with eyes open and eyes closed; backward perturbation	Coherence of acceleration between trunk and legs	Trunk-leg coherence of acceleration correlated with COP sway area; lower trunk-leg coherence of acceleration at lower frequencies in upright stance	Not performed

Table S2. Sensor-based balance evaluation in neurological disorders.

Author/Year	Participants (mean age ± SD)	Type and location of wearable sensors	Other measurement	Experimental setup	Main postural measures	Main findings	Clinical-behavioural correlations
**Multiple sclerosis**							
Sun et al. (2018) [42]	39 MS (58 ± 10)<br>15 HS (57.9 ± 13)	2 inertial sensors (1 reference accelerometer + 1 adhesive sensor patch) on L5	Force plate (COP measures)	Upright stance with eyes open and eyes closed, on firm and foam surfaces	RMS of AP and ML acceleration; 95% confidence ellipse sway area; sway path length of acceleration trajectory; mean sway velocity; total power; sway jerk	Significant correlation between measurement methods; higher sway area and total power, also depending from disease severity	Not performed
Arpan et al. (2020) [43]	25 MS (51.1 ± 2)<br>10 HS (47.6 ± 3)	6 inertial sensors (accelerometer + gyroscope) on low back, sternum, wrists and feet	Not performed	6-minute walk test	Maximum-finite-time Lyapunov exponents (local dynamic stability); dynamic stability index; distance-walked index	Similar local dynamic stability until minute 4 of walking; higher median local dynamic instability estimated over time during the test	Change in dynamic stability correlated with change in distance from minute 1 to minute 6 of walking
Chitnis et al. (2019) [44]	25 MS (46.5 ± 7) divided in 3 three severity cohorts<br>No HS	Cardiac and Activity Monitor, including an on-board IMU, on multiple body locations (upper and lower trunk, wrists, thighs and shins)	Not performed	Upright stance	Sway distance and displacement in left–right and anterior-posterior directions	Biosensor-derived metrics as reliable tools for disability monitoring in MS with respect to standard clinical evaluation	Postural sway measures correlated with MS disability (e.g. EDSS and MS functional composite-4)

Gera et al. (2020) [45]	14 MS with mild ataxia (48.6 ± 11) 11 MS with moderate ataxia (44 ± 8) 13 HS (49 ± 13)	1 inertial sensor (accelerometer + gyroscope + magnetometer) on L5	Not performed	Upright stance with eyes open and eyes closed	Sway area; jerk; path length; F95%	Higher sway area, jerk, path length and F95% in MS with moderate ataxia than MS with mild ataxia and HS	Postural sway measures negatively correlated with cerebellar white matter tract integrity
**Huntington's disease**							
Dalton et al. (2013) [46]	14 HD (51.83 ± 15) 10 HS (56.40 ± 11)	1 inertial sensor (accelerometer) on upper sternum	Not performed	Romberg test with feet together and apart	RMS of AP and ML acceleration	Higher RMS values	Not performed
Kegelmeyer et al. (2017) [47]	41 HD (52.20 ± 11) 36 HS (45.94 ± 14)	2 inertial sensors (accelerometer + gyroscope within tablet computers – iPads) on thorax and L5	Not performed	Sitting, standing and walking	Peak angular excursion; total absolute excursion; mean angular excursion	Larger peak and total excursions; abrupt changes in speed and amplitude of movements	Not significant correlations

Table S2. Sensor-based balance evaluation in neurological disorders.

Author/Year	Participants (mean age ± SD)	Type and location of wearable sensors	Other measurement	Experimental setup	Main postural measures	Main findings	Clinical-behavioural correlations
**Cerebellar ataxia**							
Van de Warrenburg et al. (2005) [48]	11 CA (49.5 ± 9) 11 HS (48.5 ± 8)	1 sensor (Sway Star system consisting of 2 digital angular-velocity transducers) on L2-L3	Not performed	Upright stance with eyes open and eyes closed, on firm and foam surfaces; walking tasks; retropulsion task; "get-up-and-go" task	Peak-to-peak excursions in trunk angular displacement and velocity, as well as trunk sway velocity, in the roll and pitch planes	Larger trunk angular displacement and velocity (pitch > roll plane) in stance, walking and retropulsion tasks	Trunk angular displacement and velocity correlated with motor impairment (e.g., ICARS scores and Tinetti's Mobility Index)

Hejda et al. (2015) [49]	10 CA (52.2 ± 12) 11 HS (26.0 ± 6)	1 IMU on L2-L3	Force plate (COP measures)	Upright stance with eyes open and eyes closed, on firm and foam surfaces	Total sway path length (roll, yaw and pitch excursions)	COP and COM measures significantly correlated; larger total sway path length in all conditions	Not performed
Kutílek et al. (2015) [50]	10 CA (52.2 ± 12) 11 HS (26.0 ± 6)	3 inertial sensors (gyroscope) on L2-L3 and feet	Not performed	Upright stance with eyes open and eyes closed, on firm and foam surfaces	Area of convex hulls of the trajectories	Larger area of convex hulls in all conditions	Not performed
Melecky et al. (2016) [51]	10 CA (52.2 ± 12) 11 HS (26.0 ± 6)	1 IMU (accelerometer + gyroscope) on L2-L3	Force plate (COP measures)	Upright stance with eyes open and eyes closed, on firm and foam surfaces	Convex polyhedron volume	COP and COM measures significantly correlated; increased convex polyhedron volume in all conditions	Not performed
Nguyen et al. (2018) [52]	34 CA (47.64 ± 11) 22 HS (not specified; age-matched)	2 inertial sensors (accelerometer) on sternum and upper-back	Not performed	Romberg and trunk test	RMS of acceleration; approximate, sample and fuzzy entropy	Greater entropy measures	Entropy measures (especially from sternum) strongly correlated with clinical assessment
Adamová et al. (2018) [53]	10 CA (52.2 ± 12) 11 HS (26.0 ± 6)	1 IMU on L2-L3	Force plate (COP measures)	Upright stance with eyes open and eyes closed, on firm and foam surfaces	Average velocity of the point; total length of the 3-dimensional trajectory	COP and COM measures significantly correlated; higher average velocity and total length only for tasks with foam surface	Not performed

Widener et al. (2020) [54]	10 CA (47.2 ± 6.6)<br>10 HS (47.8 ± 8.8)	6 IMU on lumbar spine, anterior sternum, bilateral ankles and wrists	Not performed	Modified clinical test of sensory interaction on balance, with and without weighting	95% of the ellipse sway area	Larger sway area during no-weight standing tasks; sway area generally decreased with torso weighting in CA	SARA scores correlated with standing stability measures

Table S2. Sensor-based balance evaluation in neurological disorders.

Author/Year	Participants (mean age ± SD)	Type and location of wearable sensors	Other measurement	Experimental setup	Main postural measures	Main findings	Clinical-behavioural correlations
				**Stroke**			
Perez-Cruzado et al. (2014) [55]	4 ST (76.7 ± 3)<br>No HS	2 inertial sensors on L5-S1 and T7-T8	Not performed	Single-leg stance test with eyes open and eyes closed	Displacement and velocity; rotation; flexion/extension inclination	Parameterization of single-leg test in ST; good reliability and validity of inertial sensors	Not performed
Merchan-Baeza et al. (2014) [56]	5 ST (76.7)<br>No HS	2 inertial sensors on T7 and L5-S1	Not performed	Functional reach test	Maximum angular lumbosacral/thoracic displacement; time, velocity and acceleration of displacement	Parameterization of the functional reach test in ST; excellent reliability and validity of inertial sensors	Not performed
Merchan-Baeza et al. (2015) [57]	5 ST (72.33 ± 4)<br>5 HS (73.04 ± 4)	2 inertial sensors on T7 and L5-S1	Not performed	Functional reach test	Maximum angular lumbosacral/thoracic displacement; time, velocity and acceleration of displacement	Smaller angular displacement, velocity and acceleration; higher maximum and minimum velocity and acceleration values	Not performed
Iosa et al. (2016) [58]	13 ST (63.85 ± 10)<br>10 HS (63.70 ± 8)	1 inertial sensor (accelerometer) on L2-L3	Not performed	Walking tasks	RMS of trunk acceleration	Higher trunk accelerations along the LL axis	Not performed
Rahimzadeh-Khiabani et al. (2017) [59]	12 ST with low ankle spasticity (74.3 ± 3)<br>15 ST with high ankle spasticity (61.8 ± 3)	1 IMU on lumbar region	Force plate (COP measures)	Upright stance with eyes open and eyes closed	Trunk angle, velocity and velocity frequency amplitude in	Greater trunk roll velocity and velocity frequency amplitude at 3.7 Hz and 4.9 Hz, especially with eyes	Not performed

	No HS				pitch and roll directions	closed, in ST with high ankle spasticity	
Belluscio et al. (2018) [60]	27 ST (66 ± 16)<br>18 HS (57 ± 5)	5 inertial sensors (accelerometer + gyroscope) on occipital cranium, sternum, L4-L5 and shins (lateral malleoli)	Not performed	Fukuda stepping test	RMS of AP and ML acceleration	Not significant findings	Not performed
Hou et al. (2018) [61]	10 ST (57.7 ± 13)<br>13 HS (45.6 ± 12)	1 inertial sensor (accelerometer + gyroscope within a smartphone – HTC 10) on S2	Not performed	6 standing tasks with eyes open and eyes closed, as well as with different base of support amplitudes	AP and ML acceleration change; X, Y and Z axis body tilt	Higher acceleration values, primarily during standing tasks with visual deprivation and narrow base of support	Not performed

Table S2. Sensor-based balance evaluation in neurological disorders.

Author/Year	Participants (mean age ± SD)	Type and location of wearable sensors	Other measurement	Experimental setup	Main postural measures	Main findings	Clinical-behavioural correlations
**Stroke**							
Hou et al. (2019) [62]	8 ST (52.3 ± 10)<br>8 HS (51.5 ± 9)	1 inertial sensor (accelerometer + gyroscope within a smartphone – ASUS Zenfone 3) on S2	Not performed	6 standing tasks with eyes open and eyes closed, as well as with different base of support amplitudes	AP and ML acceleration change; X, Y and Z axis body tilt (changes in angular velocity)	Greater gyroscope values in all standing tasks	Gyroscope data negatively correlated with Berg balance scale scores
**Traumatic brain injury**							
Furman et al. (2013) [63]	43 TBI (15 ± 1)<br>27 HS (16 ± 1)	1 inertial sensor (accelerometer) on pelvis	Not performed	Side-by-side and tandem upright stance, with eyes open and eyes	Normalized path length of AP acceleration	Not significant findings	Not performed

				closed, on firm and foam surfaces			
King et al. (2014) [64]	13 TBI (16.3 ± 2)<br>13 HS (16.7 ± 2)	1 inertial sensor (accelerometer) on L5	Not performed	Balance error scoring system (standard and modified version)	RMS of AP and ML acceleration	Higher RMS of AP and ML acceleration; instrumented scale more accurate than standard clinical scale in TBI identification	Not performed
King et al. (2017) [65]	52 TBI (20.36 ± 1)<br>76 HS (20.64 ± 1)	1 inertial sensor (accelerometer + gyroscope + magnetometer) on L5	Not performed	Modified balance error scoring system	132 sway metrics reflecting postural sway amplitude, velocity, variability and frequency in AP and ML directions	Higher RMS, total power, mean distance, range of acceleration and path length in ML direction, ellipse sway area, total sway area, 95% circle sway area (main measures)	Not performed
Doherty et al. (2017) [66]	15 TBI (21.83 ± 3)<br>15 HS (22.46 ± 4)	1 inertial sensor (accelerometer + gyroscope) on pelvis	Force plate (COP measures)	Bilateral, tandem and unilateral stance variants of balance error scoring system	95% ellipsoid volume of sway	Higher sway volume in bilateral stance (in accordance with increased sway area measured by force plate)	Not performed

Table S2. Sensor-based balance evaluation in neurological disorders.

Author/Year	Participants (mean age ± SD)	Type and location of wearable sensors	Other measurement	Experimental setup	Main postural measures	Main findings	Clinical-behavioural correlations
**Traumatic brain injury**							
Alkathiry et al. (2018) [67]	56 TBI (15 ± 1) No HS	1 inertial sensor (accelerometer) on lower back	Not performed	Side-by-side and tandem upright stance, with eyes open and eyes closed, on firm and foam surfaces ("balance accelerometer measure")	Normalized path length of AP sway	Greater normalized AP path length with eyes closed than eyes open, foam surface than firm surface, tandem stance than side-by-side stance	Self-reported symptoms (e.g. dizziness, headache) correlated with normalized path length of AP sway
Baracks et al. (2018) [68]	48 TBI (20.62 ± 2) 45 HS (20.85 ± 1)	1 inertial sensor (accelerometer + gyroscope + magnetometer) on L4-L5	Not performed	Bilateral, tandem and unilateral stance variants of balance error scoring system	RMS sway; 95% ellipse sway area	Higher RMS sway and 95% ellipse sway area in all stance conditions	Not performed
Gera et al. (2018) [69]	38 TBI (20.6 ± 1) 81 HS (21.0 ± 1)	1 inertial sensor (accelerometer + gyroscope + magnetometer) on L5	Not performed	Upright stance with eyes open and eyes closed, on firm and foam surfaces	Postural sway area	Larger total sway area (all conditions except upright stance with eyes open on foam surface)	Postural sway area correlated with self-reported dizziness
**Neuropathies**							
Najafi et al. (2010) [70]	17 DPN (59.2 ± 8) 21 HS (24.4 ± 2)	2 inertial sensors (accelerometer + gyroscope + magnetometer) on lower back and shin	Force plate (COP measures)	Romberg test, on firm and foam surfaces	Area of COM sway; ankle and hip sway; reciprocal compensatory index	COP and COM measures were correlated; Larger area of COM sway; higher ankle and hip sway with eyes closed; higher reciprocal index	Not performed

Toosizadeh et al. (2015) [71]	18 DPN (65 ± 8)<br>18 HS (69 ± 3)	2 inertial sensors (accelerometer + gyroscope) on lower back and shin	Not performed	Romberg test	Range of AP and ML sway; rate of body sway in short time-intervals (local-control) and long time-intervals (central control)	Higher body sway; higher local-control rate of body sway	Vibration perception threshold negatively correlated with central-control rate of body sway
D'Silva et al. (2017) [72]	14 DPN (57.4 ± 5)<br>14 HS (58.07 ± 5)	1 IMU on L3	Not performed	Upright stance with eyes open and eyes closed, on firm and foam surfaces; tandem stance with eyes open on firm surface	Range of AP and ML acceleration; AP and ML peak velocity; RMS of AP and ML acceleration	Higher AP peak velocity, AP and ML acceleration range in tandem stance, as well as in upright stance with eyes closed on foam surface; higher ML peak velocity and RMS of AP acceleration in tandem stance	Range and RMS of AP acceleration correlated with glycated haemoglobin level

Table S2. Sensor-based balance evaluation in neurological disorders.

Author/Year	Participants (mean age ± SD)	Type and location of wearable sensors	Other measurement	Experimental setup	Main postural measures	Main findings	Clinical-behavioural correlations
**Vestibular syndromes**							
Cohen et al. (2012) [73]	21 BPPV (58.8 ± 12)<br>18 AN (55.6 ± 11)<br>27 UW (54.9 ± 18)<br>61 HS (49.6 ± 16)	1 IMU on the back at the mid-thoracic	Not performed	Walking tasks (e.g. tandem gait with and without eyes open); shortened functional mobility test	RMS of acceleration and angular velocity	Higher acceleration and angular velocity in the roll and yaw axes during tandem walking with eyes closed in UW	Not performed

Kapoula et al. (2013) [74]	11 BLVF (52 ± 14) 16 HS (31.7 ± 10)	1 inertial sensor (accelerometer) on L5	Not performed	Upright stance during fixation tasks, eyes open and eyes closed	Normalized area; RMS of AP and ML sway and velocity; mean power frequency; quotient of Romberg for measures	Higher quotient of Romberg for surface area, RMS of AP sway and RMS of AP and ML velocity; during far-close-vergence higher surface area, RMS of ML sway and smaller mean power frequency; during convergence smaller surface area for both subgroups	Not performed
Kim et al. (2013) [75]	17 VN (45.6 ± 12) 18 HS (43.5 ± 15)	4 inertial sensors (accelerometer + gyroscope) on head, pelvis and legs	Not performed	Modified Romberg test on foam surface	Signal vector magnitude; angular velocity	Greater signal vector magnitude; bigger group difference with signal vector magnitude than angular velocity, as well as with head sensor than pelvis and legs sensors	Not performed
D'Silva et al. (2017) [72]	13 BPPV (54.5 ± 6) 11 BPPVDM (57.6 ± 6) 14 HS (58.07 ± 5)	1 IMU on L3	Not performed	Upright stance with eyes open and eyes closed, on firm and foam surfaces; tandem stance with eyes open on firm surface	Range of AP and ML acceleration; AP and ML peak velocity; RMS of AP and ML acceleration	Higher range of AP-ML acceleration, AP-ML peak velocity and RMS of AP acceleration in BPPVDM than HS (several conditions); higher AP-ML peak velocity in BPPVDM than BPPV (eyes	Range and RMS of AP acceleration correlated with glycated haemoglobin level in BPPVDM

	closed, foam surface)

ABC: Activities-Specific Balance Confidence scale; AN: patients with Acoustic Neuroma; AP: Antero-Posterior; APAs: Anticipatory Postural Adjustments; BLVF: patients with Bilateral Loss of Vestibular Function; BPPV: patients with Benign Paroxysmal Positional Vertigo; BPPVDM: patients with Benign Paroxysmal Positional Vertigo and Diabetes Mellitus; CA: Cerebellar Ataxia; COM: Center Of Mass; COP: Center Of Pressure; DPN: patients with Diabetic Peripheral Neuropathy; F95%: frequency comprising 95% of the signal; EDSS: Expanded Disability Status Scale; FOG: Freezing of Gait; HD: Huntington's Disease; HS: Healthy Subjects; ICARS: International Cooperative Ataxia Rating Scale; IMU: Inertial Measurement Unit; ML: Medio-Lateral; MS: patients with Multiple Sclerosis; MSWS12: 12-Item Multiple Sclerosis Walking Scale; OFF state of therapy: not under dopaminergic therapy; OLS: One-Leg Stance; ON state of therapy: under dopaminergic therapy; PD: patients with Parkinson's Disease; PIGD: Postural Instability Gait Difficulty; RMS: Root Mean Square; SARA: Scale for the Assessment and Rating of Ataxia; sEMG: Surface Electromyography; SOT: Sensory Organization Test;: ST: patients with a previous Stroke; TBI: patients with Traumatic Brain Injury; UPDRS-III: Unified Parkinson's Disease Rating Scale – part III; UW: patients with unilateral vestibular weakness; VN: patients with Vestibular Neuritis

References

1. Díaz, S.; Stephenson, J.B.; Labrador, M.A. Use of wearable sensor technology in gait, balance, and range of motion analysis. *Appl. Sci.* **2020**, *10*, 234.
2. Ghislieri, M.; Gastaldi, L.; Pastorelli, S.; Tadano, S.; Agostini, V. Wearable Inertial Sensors to Assess Standing Balance: A Systematic Review. *Sensors* **2019**, *19*, 4075, doi:10.3390/s19194075.
3. Pinho, A.S.; Salazar, A.P.; Hennig, E.M.; Spessato, B.C.; Domingo, A.; Pagnussat, A.S. Can we rely on mobile devices and other gadgets to assess the postural balance of healthy individuals? A systematic review. *Sensors* **2019**, *19*, 2972, doi:10.3390/s19132972.
4. Pang, I.; Okubo, Y.; Sturnieks, D.; Lord, S.R.; Brodie, M.A. Detection of Near Falls Using Wearable Devices: A Systematic Review. *J. Geriatr. Phys. Ther.* **2019**, *42*, 48–56.
5. Moral-Munoz, J.A.; Esteban-Moreno, B.; Herrera-Viedma, E.; Cobo, M.J.; Pérez, I.J. Smartphone Applications to Perform Body Balance Assessment: A Standardized Review. *J. Med Syst.* **2018**, *42*, 1–8.
6. Sun, R.; McGinnis, R.; Sosnoff, J.J. Novel technology for mobility and balance tracking in patients with multiple sclerosis: A systematic review. *Expert Rev. Neurother.* **2018**, *18*, 887–898.
7. Sun, R.; Sosnoff, J.J. Novel sensing technology in fall risk assessment in older adults: A systematic review. *BMC Geriatr.* **2018**, *18*, 14.
8. Gordt, K.; Gerhardy, T.; Najafi, B.; Schwenk, M. Effects of Wearable Sensor-Based Balance and Gait Training on Balance, Gait, and Functional Performance in Healthy and Patient Populations: A Systematic Review and Meta-Analysis of Randomized Controlled Trials. *Gerontology* **2017**, *64*, 74–89, doi:10.1159/000481454.
9. Roeing, K.L.; Hsieh, K.L.; Sosnoff, J.J. A systematic review of balance and fall risk assessments with mobile phone technology. *Arch. Gerontol. Geriatr.* **2017**, *73*, 222–226.
10. Godinho, C.; Domingos, J.; Cunha, G.; Santos, A.T.; Fernandes, R.M.; Abreu, D.; Gonçalves, N.; Matthews, H.; Isaacs, T.; Duffen, J.; et al. A systematic review of the characteristics and validity of monitoring technologies to assess Parkinson's disease. *J. Neuroeng. Rehabil.* **2016**, *13*, 24, doi:10.1186/s12984-016-0136-7.
11. Ma, C.Z.H.; Wong, D.W.C.; Lam, W.K.; Wan, A.H.P.; Lee, W.C.C. Balance improvement effects of biofeedback systems with state-of-the-art wearable sensors: A systematic review. *Sensors* **2016**, *16*, 434, doi:10.3390/s16040434.
12. Hubble, R.P.; Naughton, G.A.; Silburn, P.A.; Cole, M.H. Wearable sensor use for assessing standing balance and walking stability in people with Parkinson's disease: A systematic review. *PLoS ONE* **2015**, *10*, e0123705, doi:10.1371/journal.pone.0123705.
13. Maetzler, W.; Domingos, J.; Srulijes, K.; Ferreira, J.J.; Bloem, B.R. Quantitative wearable sensors for objective assessment of Parkinson's disease. *Mov. Disord.* **2013**, *28*, 1628–1637.
14. Howcroft, J.; Kofman, J.; Lemaire, E.D. Review of fall risk assessment in geriatric populations using inertial sensors. *J. Neuroeng. Rehabil.* **2013**, *10*, 1–12.
15. Gago, M.F.; Fernandes, V.; Ferreira, J.; Silva, H.; Rocha, L.; Bicho, E.; Sousa, N. Postural Stability Analysis with Inertial Measurement Units in Alzheimer's Disease. *Dement. Geriatr. Cognit. Dis. Extra* **2014**, *4*, 22–30, doi:10.1159/000357472.
16. Hsu, Y.L.; Chung, P.C.J.; Wang, W.H.; Pai, M.C.; Wang, C.Y.; Lin, C.W.; Wu, H.L.; Wang, J.S. Gait and balance analysis for patients with Alzheimer's disease using an inertial-sensor-based wearable instrument. *IEEE J. Biomed. Health Inform.* **2014**, *18*, 1822–1830, doi:10.1109/JBHI.2014.2325413.
17. Gago, M.F.; Yelshyna, D.; Bicho, E.; Silva, H.D.; Rocha, L.; Lurdes Rodrigues, M.; Sousa, N. Compensatory Postural Adjustments in an Oculus Virtual Reality Environment and the Risk of Falling in Alzheimer's Disease. *Dement. Geriatr. Cogniy. Dis. Extra* **2016**, *6*, 252–267, doi:10.1159/000447124
18. Mancini, M.; Zampieri, C.; Carlson-Kuhta, P.; Chiari, L.; Horak, F.B. Anticipatory postural adjustments prior to step initiation are hypometric in untreated Parkinson's disease: An accelerometer-based approach. *Eur. J. Neurol.* **2009**, *16*, 1028–1034, doi:10.1111/j.1468-1331.2009.02641.x.
19. Mancini, M.; Horak, F.B.; Zampieri, C.; Carlson-Kuhta, P.; Nutt, J.G.; Chiari, L. Trunk accelerometry reveals postural instability in untreated Parkinson's disease. *Parkinsonism Relat. Disord.* **2011**, *17*, 557–562, doi:10.1016/j.parkreldis.2011.05.010.
20. Mancini, M.; Salarian, A.; Carlson-Kuhta, P.; Zampieri, C.; King, L.; Chiari, L.; Horak, F.B. ISway: A sensitive, valid and reliable measure of postural control. *J. Neuroeng. Rehabil.* **2012**, *9*, 59, doi:10.1186/1743-0003-9-59.
21. Maetzler, W.; Mancini, M.; Liepelt-Scarfone, I.; Müller, K.; Becker, C.; van Lummel, R.C.; Ainsworth, E.; Hobert, M.; Streffer, J.; Berg, D.; et al. Impaired trunk stability in individuals at high risk for Parkinson's disease. *PLoS ONE* **2012**, *7*, e32240, doi:10.1371/journal.pone.0032240.

22. Baston, C.; Mancini, M.; Schoneburg, B.; Horak, F.; Rocchi, L. Postural strategies assessed with inertial sensors in healthy and parkinsonian subjects. *Gait Posture* **2014**, *40*, 70–75, doi:10.1016/j.gaitpost.2014.02.012.
23. Curtze, C.; Nutt, J.G.; Carlson-Kuhta, P.; Mancini, M.; Horak, F.B. Objective Gait and Balance Impairments Relate to Balance Confidence and Perceived Mobility in People with Parkinson Disease. *Phys. Ther.* **2016**, *96*, 1734–1743, doi:10.2522/ptj.20150662.
24. Mancini, M.; Chiari, L.; Holmstrom, L.; Salarian, A.; Horak, F.B. Validity and reliability of an IMU-based method to detect APAs prior to gait initiation. *Gait Posture* **2016**, *43*, 125–131, doi:10.1016/j.gaitpost.2015.08.015.
25. Baston, C.; Mancini, M.; Rocchi, L.; Horak, F. Effects of Levodopa on Postural Strategies in Parkinson's disease. *Gait Posture* **2016**, *46*, 26–29, doi:10.1016/j.gaitpost.2016.02.009.
26. Falaki, A.; Huang, X.; Lewis, M.M.; Latash, M.L. Impaired Synergic Control of Posture in Parkinson's Patients without Postural Instability. *Gait Posture* **2016**, *44*, 209–215, doi:10.1016/j.gaitpost.2015.12.035.
27. Falaki, A.; Huang, X.; Lewis, M.M.; Latash, M.L. Dopaminergic modulation of multi-muscle synergies in postural tasks performed by patients with Parkinson's disease. *J. Electromyogr. Kinesiol.* **2017**, *33*, 20–26, doi:10.1016/j.jelekin.2017.01.002.
28. De Souza Fortaleza, A.C.; Mancini, M.; Carlson-Kuhta, P.; King, L.A.; Nutt, J.G.; Chagas, E.F.; Freitas, I.F.; Horak, F.B. Dual task interference on postural sway, postural transitions and gait in people with Parkinson's disease and freezing of gait. *Gait Posture* **2017**, *56*, 76–81, doi:10.1016/j.gaitpost.2017.05.006.
29. Ozinga, S.J.; Linder, S.M.; Alberts, J.L. Use of Mobile Device Accelerometry to Enhance Evaluation of Postural Instability in Parkinson Disease. *Arch. Phys. Med. Rehabil.* **2017**, *98*, 649–658, doi:10.1016/j.apmr.2016.08.479.
30. Ozinga, S.J.; Koop, M.M.; Linder, S.M.; Machado, A.G.; Dey, T.; Alberts, J.L. Three-dimensional evaluation of postural stability in Parkinson's disease with mobile technology. *NeuroRehabilitation* **2017**, *41*, 211–218, doi:10.3233/NRE-171473.
31. Bonora, G.; Mancini, M.; Carpinella, I.; Chiari, L.; Ferrarin, M.; Nutt, J.G.; Horak, F.B. Investigation of Anticipatory Postural Adjustments during One-Leg Stance Using Inertial Sensors: Evidence from Subjects with Parkinsonism. *Front. Neurol.* **2017**, *8*, 361, doi:10.3389/fneur.2017.00361.
32. Bonora, G.; Mancini, M.; Carpinella, I.; Chiari, L.; Horak, F.B.; Ferrarin, M. Gait initiation is impaired in subjects with Parkinson's disease in the OFF state: Evidence from the analysis of the anticipatory postural adjustments through wearable inertial sensors. *Gait Posture* **2017**, *51*, 218–221, doi:10.1016/j.gaitpost.2016.10.017.
33. Chen, T.; Fan, Y.; Zhuang, X.; Feng, D.; Chen, Y.; Chan, P.; Du, Y. Postural sway in patients with early Parkinson's disease performing cognitive tasks while standing. *Neurol. Res.* **2018**, *40*, 491–498, doi:10.1080/01616412.2018.1451017.
34. Lang, K.C.; Hackney, M.E.; Ting, L.H.; McKay, J.L. Antagonist muscle activity during reactive balance responses is elevated in Parkinson's disease and in balance impairment. *PLoS ONE* **2019**, *14*, e0211137, doi:10.1371/journal.pone.0211137.
35. Spain, R.I.; St George, R.J.; Salarian, A.; Mancini, M.; Wagner, J.M.; Horak, F.B.; Bourdette, D. Body-worn motion sensors detect balance and gait deficits in people with multiple sclerosis who have normal walking speed. *Gait Posture* **2012**, *35*, 573–578, doi:10.1016/j.gaitpost.2011.11.026.
36. Spain, R.I.; Mancini, M.; Horak, F.B.; Bourdette, D. Body-worn sensors capture variability, but not decline, of gait and balance measures in multiple sclerosis over 18 months. *Gait Posture* **2014**, *39*, 958–964, doi:10.1016/j.gaitpost.2013.12.010.
37. Solomon, A.J.; Jacobs, J.V.; Lomond, K.V.; Henry, S.M. Detection of postural sway abnormalities by wireless inertial sensors in minimally disabled patients with multiple sclerosis: A case-control study. *J. Neuroeng. Rehabil.* **2015**, *12*, 74, doi:10.1186/s12984-015-0066-9.
38. Craig, J.J.; Bruetsch, A.P.; Lynch, S.G.; Horak, F.B.; Huisinga, J.M. Instrumented balance and walking assessments in persons with multiple sclerosis show strong test-retest reliability. *J. Neuroeng. Rehabil.* **2017**, *14*, 43, doi:10.1186/s12984-017-0251-0.
39. El-Gohary, M.; Peterson, D.; Gera, G.; Horak, F.B.; Huisinga, J.M. Validity of the Instrumented Push and Release Test to Quantify Postural Responses in Persons With Multiple Sclerosis. *Arch. Phys. Med. Rehabil.* **2017**, *98*, 1325–1331, doi:10.1016/j.apmr.2017.01.030.

40. Witchel, H.J.; Oberndorfer, C.; Needham, R.; Healy, A.; Westling, C.E.I.; Guppy, J.H.; Bush, J.; Barth, J.; Herberz, C.; Roggen, D.; et al. Thigh-Derived Inertial Sensor Metrics to Assess the Sit-to-Stand and Stand-to-Sit Transitions in the Timed Up and Go (TUG) Task for Quantifying Mobility Impairment in Multiple Sclerosis. *Front. Neurol.* **2018**, *9*, 684, doi:10.3389/fneur.2018.00684.
41. Huisinga, J.; Mancini, M.; Veys, C.; Spain, R.; Horak, F. Coherence analysis of trunk and leg acceleration reveals altered postural sway strategy during standing in persons with multiple sclerosis. *Hum. Mov. Sci.* **2018**, *58*, 330–336, doi:10.1016/j.humov.2017.12.009.
42. Sun, R.; Moon, Y.; McGinnis, R.S.; Seagers, K.; Motl, R.W.; Sheth, N.; Wright, J.A.; Ghaffari, R.; Patel, S.; Sosnoff, J.J. Assessment of Postural Sway in Individuals with Multiple Sclerosis Using a Novel Wearable Inertial Sensor. *Digit. Biomark.* **2018**, *2*, 1–10, doi:10.1159/000485958.
43. Arpan, I.; Fino, P.C.; Fling, B.W.; Horak, F. Local dynamic stability during long-fatiguing walks in people with multiple sclerosis. *Gait Posture* **2020**, *76*, 122–127, doi:10.1016/j.gaitpost.2019.10.032.
44. Chitnis, T.; Glanz, B.I.; Gonzalez, C.; Healy, B.C.; Saraceno, T.J.; Sattarnezhad, N.; Diaz-Cruz, C.; Polgar-Turcsanyi, M.; Tummala, S.; Bakshi, R.; et al. Quantifying neurologic disease using biosensor measurements in-clinic and in free-living settings in multiple sclerosis. *NPJ Digit. Med.* **2019**, *2*, 123, doi:10.1038/s41746-019-0197-7.
45. Gera, G.; Fling, B.W.; Horak, F.B. Cerebellar White Matter Damage Is Associated with Postural Sway Deficits in People with Multiple Sclerosis. *Arch. Phys. Med. Rehabil.* **2020**, *101*, 258–264, doi:10.1016/j.apmr.2019.07.011.
46. Dalton, A.; Khalil, H.; Busse, M.; Rosser, A.; van Deursen, R.; Ólaighin, G. Analysis of gait and balance through a single triaxial accelerometer in presymptomatic and symptomatic Huntington's disease. *Gait Posture* **2013**, *37*, 49–54, doi:10.1016/j.gaitpost.2012.05.028.
47. Kegelmeyer, D.A.; Kostyk, S.K.; Fritz, N.E.; Fiumedora, M.M.; Chaudhari, A.; Palettas, M.; Young, G.; Kloos, A.D. Quantitative biomechanical assessment of trunk control in Huntington's disease reveals more impairment in static than dynamic tasks. *J. Neurol. Sci.* **2017**, *376*, 29–34, doi:10.1016/j.jns.2017.02.054.
48. Van de Warrenburg, B.P.C.; Bakker, M.; Kremer, B.P.H.; Bloem, B.R.; Allum, J.H.J. Trunk sway in patients with spinocerebellar ataxia. *Mov. Disord.* **2005**, *20*, 1006–1013, doi:10.1002/mds.20486.
49. Hejda, J.; Cakrt, O.; Socha, V.; Schlenker, J.; Kutilek, P. 3-D trajectory of body sway angles: A technique for quantifying postural stability. *Biocybern. Biomed. Eng.* **2015**, *35*, 185–191, doi:10.1016/j.bbe.2015.02.001.
50. Kutílek, P.; Socha, V.; Čakrt, O.; Svoboda, Z. Assessment of postural stability in patients with cerebellar disease using gyroscope data. *J. Bodyw. Mov. Ther.* **2015**, *19*, 421–428, doi:10.1016/j.jbmt.2014.09.005.
51. Melecky, R.; Socha, V.; Kutilek, P.; Hanakova, L.; Takac, P.; Schlenker, J.; Svoboda, Z. Quantification of Trunk Postural Stability Using Convex Polyhedron of the Time-Series Accelerometer Data. *J. Healthc. Eng.* **2016**, *2016*, doi:10.1155/2016/1621562.
52. Nguyen, N.; Phan, D.; Pathirana, P.N.; Horne, M.; Power, L.; Szmulewicz, D. Quantification of Axial Abnormality Due to Cerebellar Ataxia with Inertial Measurements. *Sensors* **2018**, *18*, 2791, doi:10.3390/s18092791.
53. Adamová, B.; Kutilek, P.; Cakrt, O.; Svoboda, Z.; Viteckova, S.; Smrcka, P. Quantifying postural stability of patients with cerebellar disorder during quiet stance using three-axis accelerometer. *Biomed. Signal Process. Control* **2018**, *40*, 378–384, doi:10.1016/j.bspc.2017.09.025.
54. Widener, G.L.; Conley, N.; Whiteford, S.; Gee, J.; Harrell, A.; Gibson-Horn, C.; Block, V.; Allen, D.D. Changes in standing stability with balance-based torso-weighting with cerebellar ataxia: A pilot study. *Physiother. Res. Int.* **2020**, *25*, e1814, doi:10.1002/pri.1814.
55. Perez-Cruzado, D.; González-Sánchez, M.; Cuesta-Vargas, A.I. Parameterization and reliability of single-leg balance test assessed with inertial sensors in stroke survivors: A cross-sectional study. *Biomed. Eng. Online* **2014**, *13*, 127, doi:10.1186/1475-925X-13-127.
56. Merchán-Baeza, J.A.; González-Sánchez, M.; Cuesta-Vargas, A.I. Reliability in the parameterization of the functional reach test in elderly stroke patients: A pilot study. *Biomed. Res. Int.* **2014**, *2014*, 637671, doi:10.1155/2014/637671.
57. Merchán-Baeza, J.A.; González-Sánchez, M.; Cuesta-Vargas, A.I. Comparison of kinematic variables obtained by inertial sensors among stroke survivors and healthy older adults in the Functional Reach Test: Cross-sectional study. *Biomed. Eng. Online* **2015**, *14*, 49, doi:10.1186/s12938-015-0047-z.
58. Iosa, M.; Bini, F.; Marinozzi, F.; Fusco, A.; Morone, G.; Koch, G.; Martino Cinnera, A.; Bonnì, S.; Paolucci, S. Stability and Harmony of Gait in Patients with Subacute Stroke. *J. Med. Biol. Eng.* **2016**, *36*, 635–643, doi:10.1007/s40846-016-0178-0.

59. Rahimzadeh Khiabani, R.; Mochizuki, G.; Ismail, F.; Boulias, C.; Phadke, C.P.; Gage, W.H. Impact of Spasticity on Balance Control during Quiet Standing in Persons after Stroke. *Stroke Res. Treat.* **2017**, *2017*, 6153714, doi:10.1155/2017/6153714.
60. Belluscio, V.; Bergamini, E.; Iosa, M.; Tramontano, M.; Morone, G.; Vannozzi, G. The iFST: An instrumented version of the Fukuda Stepping Test for balance assessment. *Gait Posture* **2018**, *60*, 203–208, doi:10.1016/j.gaitpost.2017.12.010.
61. Hou, Y.R.; Chiu, Y.L.; Chiang, S.L.; Chen, H.Y.; Sung, W.H. Feasibility of a smartphone-based balance assessment system for subjects with chronic stroke. *Comput. Methods Programs Biomed.* **2018**, *161*, 191–195, doi:10.1016/j.cmpb.2018.04.027.
62. Hou, Y.R.; Chiu, Y.L.; Chiang, S.L.; Chen, H.Y.; Sung, W.H. Development of a Smartphone-Based Balance Assessment System for Subjects with Stroke. *Sensors* **2019**, *20*, 88, doi:10.3390/s20010088.
63. Furman, G.R.; Lin, C.C.; Bellanca, J.L.; Marchetti, G.F.; Collins, M.W.; Whitney, S.L. Comparison of the balance accelerometer measure and balance error scoring system in adolescent concussions in sports. *Am. J. Sports Med.* **2013**, *41*, 1404–1410, doi:10.1177/0363546513484446.
64. King, L.A.; Horak, F.B.; Mancini, M.; Pierce, D.; Priest, K.C.; Chesnutt, J.; Sullivan, P.; Chapman, J.C. Instrumenting the Balance Error Scoring System for use with patients reporting persistent balance problems after mild traumatic brain injury. *Arch. Phys. Med. Rehabil.* **2014**, *95*, 353–359, doi:10.1016/j.apmr.2013.10.015.
65. King, L.A.; Mancini, M.; Fino, P.C.; Chesnutt, J.; Swanson, C.W.; Markwardt, S.; Chapman, J.C. Sensor-Based Balance Measures Outperform Modified Balance Error Scoring System in Identifying Acute Concussion. *Ann. Biomed. Eng.* **2018**, *45*, 2135–2145, doi:10.1007/s10439-017-1856-y.
66. Doherty, C.; Zhao, L.; Ryan, J.; Komaba, Y.; Inomata, A.; Caulfield, B. Quantification of postural control deficits in patients with recent concussion: An inertial-sensor based approach. *Clin. Biomech.* **2017**, *42*, 79–84, doi:10.1016/j.clinbiomech.2017.01.007.
67. Alkathiry, A.A.; Sparto, P.J.; Freund, B.; Whitney, S.L.; Mucha, A.; Furman, J.M.; Collins, M.W.; Kontos, A.P. Using Accelerometers to Record Postural Sway in Adolescents With Concussion: A Cross-Sectional Study. *J. Athl. Train.* **2018**, *53*, 1166–1172, doi:10.4085/1062-6050-518-17.
68. Baracks, J.; Casa, D.J.; Covassin, T.; Sacko, R.; Scarneo, S.E.; Schnyer, D.; Yeargin, S.W.; Neville, C. Acute Sport-Related Concussion Screening for Collegiate Athletes Using an Instrumented Balance Assessment. *J. Athl. Train.* **2018**, *53*, 597–605, doi:10.4085/1062-6050-174-17.
69. Gera, G.; Chesnutt, J.; Mancini, M.; Horak, F.B.; King, L.A. Inertial Sensor-Based Assessment of Central Sensory Integration for Balance After Mild Traumatic Brain Injury. *Mil. Med.* **2018**, *183*, 327–332, doi:10.1093/milmed/usx162.
70. Najafi, B.; Horn, D.; Marclay, S.; Crews, R.T.; Wu, S.; Wrobel, J.S. Assessing postural control and postural control strategy in diabetes patients using innovative and wearable technology. *J. Diabetes Sci. Technol.* **2010**, *4*, 780–791, doi:10.1177/193229681000400403.
71. Toosizadeh, N.; Mohler, J.; Armstrong, D.G.; Talal, T.K.; Najafi, B. The influence of diabetic peripheral neuropathy on local postural muscle and central sensory feedback balance control. *PLoS ONE* **2015**, *10*, e0135255, doi:10.1371/journal.pone.0135255.
72. D'Silva, L.J.; Kluding, P.M.; Whitney, S.L.; Dai, H.; Santos, M. Postural sway in individuals with type 2 diabetes and concurrent benign paroxysmal positional vertigo. *Int. J. Neurosci.* **2017**, *127*, 1065–1073, doi:10.1080/00207454.2017.1317249.
73. Cohen, H.S.; Mulavara, A.P.; Peters, B.T.; Sangi-Haghpeykar, H.; Bloomberg, J.J. Tests of walking balance for screening vestibular disorders. *J. Vestib. Res.* **2012**, *22*, 95–104, doi:10.3233/VES-2012-0443.
74. Kapoula, Z.; Gaertner, C.; Yang, Q.; Denise, P.; Toupet, M. Vergence and Standing Balance in Subjects with Idiopathic Bilateral Loss of Vestibular Function. *PLoS ONE* **2013**, *8*, e66652, doi:10.1371/journal.pone.0066652.
75. Kim, S.C.; Kim, M.J.; Kim, N.; Hwang, J.H.; Han, G.C. Ambulatory balance monitoring using a wireless attachable three-axis accelerometer. *J. Vestib. Res.* **2013**, *23*, 217–225, doi:10.3233/VES-130489.
